# A clinical prediction model identifies a subgroup with inferior survival within intermediate risk acute myeloid leukemia

**DOI:** 10.7150/jca.57231

**Published:** 2021-06-11

**Authors:** Xiaoxia Hu, Bianhong Wang, Qi Chen, Aijie Huang, Weijia Fu, Lixia Liu, Ying Zhang, Gusheng Tang, Hui Cheng, Xiong Ni, Lei Gao, Jie Chen, Li Chen, Weiping Zhang, Jianmin Yang, Shanbo Cao, Li Yu, Jianmin Wang

**Affiliations:** 1 Department of Hematology, Institute of Hematology, Changhai Hospital, Shanghai 200433, China.; 2 Department of Hematology, Beijing Tsinghua Changgung Hospital, School of Clinical Medicine, Tsinghua University, Beijing 102218, China.; 3 Department of Hematology, Chinese PLA General Hospital, Beijing, 100853, China.; 4 Department of Health Statistics, Second Military Medical University, Shanghai 200433, China.; 5 Acornmed Biotechnology Co., Ltd. Beijing, 100176, China.; 6 Department of Hematology and Oncology, Shenzhen University General Hospital; Shenzhen University International Cancer Center, Shenzhen University Health Science Center, Shenzhen, 518000, China.

**Keywords:** Acute myeloid leukemia, Intermediate risk, Nomogram, Prediction model, allogenic hematopoietic stem cell transplantation

## Abstract

Intermediate risk acute myeloid leukemia (AML) comprises around 50% of AML patients and is featured with heterogeneous clinical outcomes. The study aimed to generate a prediction model to identify intermediate risk AML patients with an inferior survival. We performed targeted next generation sequencing analysis for 121 patients with 2017 European LeukemiaNet-defined intermediate risk AML, revealing 122 mutated genes, with 24 genes mutated in > 10% of patients. A prognostic nomogram characterized by white blood cell count ≥10×10^9^/L at diagnosis, mutated *DNMT3A* and genes involved in signaling pathways was developed for 110 patients who were with clinical outcomes. Two subgroups were identified: intermediate low risk (ILR; 43.6%, 48/110) and intermediate high risk (IHR; 56.4%, 62/110). The model was prognostic of overall survival (OS) and relapse-free survival (RFS) (OS: Concordance index [C-index]: 0.703, 95%CI: 0.643-0.763; RFS: C-index: 0.681, 95%CI 0.620-0.741), and was successfully validated with two independent cohorts. Allogeneic hematopoietic stem cell transplantation (alloHSCT) reduced the relapse risk of IHR patients (3-year RFS: alloHSCT: 40.0±12.8% *vs.* chemotherapy: 8.6±5.8%, *P*= 0.010). The prediction model can help identify patients with an unfavorable prognosis and refine risk-adapted therapy for intermediate risk AML patients.

## Introduction

Acute myeloid leukemia (AML) is a heterogeneous disease featured with impaired differentiation and uncontrolled proliferation of myeloid progenitors, accompanied by the suppression of normal hematopoietic cells 1-4. For several years, cytogenetics has been used for AML risk classification 5-7. With the advent of next generation sequencing (NGS), numerous mutated somatic genes have been identified in AML 8-10. In 2010, the European LeukemiaNet (ELN) incorporated the cytogenetic and molecular characteristics and proposed a classification dividing AML patients into favorable, intermediate, and adverse risk groups, and was revised in 2017 11, 12. However, this classification does not completely reflect the heterogeneity within each subgroup, in particular, the biology and prognosis of patients in the “intermediate risk AML” group are highly different. Numerous studies have attempted to improve risk stratification 13-22. A study from Karolinska Institute validated six of most promising molecular-based models for AML risk stratification 17-21. As expected, these models combining molecular and clinical data added prognostic value to the current risk classification 23.

The more recent 2017 ELN AML classification includes molecular data, however, the mutation data is restricted to *NPM1*, *CEBPA*, *TP53*, *ASXL1*, *RUNX1* and *FLT3-ITD*. Other gene mutations also have predictive values for prognosis. For instance, *DNMT3A* mutation can serve as a poor prognostic factor in AML 24, 25. In an intermediate risk AML cohort treated at Cleveland Clinic, *DNMT3A*, *U2AF1* and *EZH2* mutations were important prognostic factors for overall survival (OS) and relapse-free survival (RFS) 26. Hou *et al.* re-stratified 229 AML patients with intermediate cytogenetics into three groups with distinct prognoses based on an 8-gene mutation panel including *DNMT3A* 27.

Appropriate post-remission therapy (PRT) for intermediate risk AML remains inconclusive 28-30. We previously reported that allogeneic hematopoietic stem cell transplantation (alloHSCT) was a preferable treatment for intermediate risk AML patients 28. However, long-term survival is discounted by non-relapse mortality (NRM). To select the patients who would truly benefit from alloHSCT at early stage of the disease is the goal of risk stratification. In this study, we developed and validated a prediction model that combined clinical and molecular profiles. The model improved risk stratification by dividing intermediate risk patients into two groups with distinct prognosis and identified patients for whom alloHSCT significantly reduced relapse rates and prolonged survival.

## Materials and methods

### Patients

In total, 265 newly diagnosed AML patients (excluding acute promyelocytic leukemia), who were diagnosed and treated in Changhai Hospital from January 2010 to January 2019, were included, and patients who were with Eastern Cooperative Oncology Group (ECOG) performance status >3 (n=3), died from any reasons before or during induction (n=4), without bone marrow samples at diagnosis (n=2) were excluded. At diagnosis, the leukemic DNA of all patients was sampled and cryopreserved for mutation analysis. The median follow-up duration was 34 months (range: 1-114 months). The last follow-up was May 31, 2019.

According to the ELN criteria 12, 62 patients were classified as having a favorable risk (62/265, 23.4%), 127 patients with an intermediate risk (47.9%), and 76 patients with adverse risk (28.7%). Among patients with intermediate risk AML, 121 patients were aged ≤ 65 years (Table 1). To build a prediction model, 110 of 121 patients with clinical outcomes were included (Supplementary Figure S1). Patients with a favorable risk had better survival outcomes than those with an intermediate and adverse risk, respectively (Supplementary Figures S2A-2B). For external validation, we used two independent intermediate risk AML cohorts. Cohort 1 was ELN intermediate risk AML patients from The Cancer Genome Atlas (TCGA), who aged between 14 and 65 and treated with intensive therapy (n=41). Because the relapse data were not available in TCGA database, we used OS data of cohort 1 herein. Cohort 2 was patients aged 14-65 years with ELN-defined intermediate risk AML from Chinese PLA General Hospital (n=99, Supplementary Table S1).

Institutional databases were retrospectively reviewed to extract demographic, clinical and genetic data. All procedures complied with the tenets of the Helsinki Declaration, and approved by respective Institutional Review Boards. The requirement for written informed consent was waived, owing to the non-interventional and retrospective nature of the study.

### High-throughput sequencing, data processing and variant calling workflow sequencing

Genomic DNA was extracted from bone marrow samples of the patients at diagnosis using a customized kit, and library amplification was performed on the basis of the KAPA Hyper Prep Kit, and a 210-gene panel (Acornmed Biotechnology, Supplementary Table S2) was used to capture the target region. Multiplexed libraries were sequenced using 150-bp paired-end runs on Illumina Novaseq. To ensure the data quality, the following criteria were applied to filter the raw variant results: average effective sequencing depth of ≥1,000×on target per sample; allele mutation frequency ≥0.5% for a single nucleotide variation, and insertion or deletion; all reads were filtered with a high mapping quality (≥30) and base quality (≥30); the mutant reads were confirmed in the positive and negative strands.

### Bioinformatics analysis

Pre-processing of raw sequences and quality control statistics were performed using an in-house QC package for parameter optimization using FastP (0.19.3). The reads were aligned to the hg19 version of the human genome using the Burrows-Wheeler Alignment tool (BWA, version 0.7.12). PCR duplicates were marked using the MarkDuplicates tool in Picard. IndelRealigner and BaseRecalibrator from the Genome Analysis Toolkit (GATK, version 3.8) were used to realign and recalibrate the results of BWA alignment, respectively. Mutect2 was used to identify the variant calling of single nucleotide variants (SNVs), and insertions or deletions (INDELs). Candidate variants were obtained through background database filtering of normal samples. Pindel was used to detect *FLT3-ITD*. Quantitative analysis of *FLT3-ITD* was performed using in-house tools based on machine learning. All variants were annotated using the ANNOVAR software. These variants were further filtered to exclude synonymous variants, as well as SNVs listed as SNPs in the 1000 Genomes Project database (Oct 2014 release), dbSNP142 or our in-house SNP database, but were not reported in COSMIC as hematopoietic or somatic mutations.

### Treatment protocols

The treatment protocols were previously reported 28. In brief, patients achieving complete remission (CR) received four cycles of consolidation with cytarabine (PR-CT group) or proceeded to alloHSCT (alloHSCT group) after at least 2 cycles of consolidation, when a matching donor was available. In the alloHSCT group, patients were conditioned with the myeloablative regimen as previously reported 28, 31. Anti-thymocyte globulin was administered to patients with an unrelated or mismatched related donor. All transplanted patients received cyclosporine, short-term methotrexate, and mycophenolate mofetil as prophylaxis for graft versus host disease 32.

### Definitions

Hematological CR was defined as the presence of <5% of blasts in the bone marrow, the absence of extramedullary disease, an absolute neutrophil count of >1.0×10^9^/L, a platelet (PLT) count of >100×10^9^/L, and independence from red blood cell transfusions. Relapse was defined as the presence of ≥5% blasts in the bone marrow aspirate or peripheral blood, or the presence of extramedullary disease. OS was calculated from the date of the diagnosis until death or the last follow-up, while RFS was measured from the date of CR1 until death, the first relapse, or the last follow-up in continuous CR.

### Statistical analysis

Clinical characteristics across groups were compared using the chi-square or a two-sided Fisher's exact test for categorical variables, and the t-test or nonparametric test was used for continuous variables. The Kaplan-Meier method was used to estimate OS and RFS. The log-rank test was performed to compare the survival curves. As the median time from diagnosis to alloHSCT was 123 days, a four-month landmark was set for OS in training cohort. For RFS, as the median time from CR to alloHSCT was 96 days, a three-month landmark was set. Correspondingly, three-month and two-month landmarks were set for OS and RFS in cohort 2, respectively. (Supplementary Table S1) Cumulative incidence of relapse (CIR) and NRM were compared between groups as described previously by Gray with estimates reported by Kalbfleisch and Prentice 33.

Next, we used the Least Absolute Shrinkage and Selector Operation (LASSO) Cox regression model to determine prognostic factors from the variables with *P*<0.05 in the log-rank tests, which is a robust method widely used to select variables and generate a predictive model 34-36. The penalty parameter λ controls the amount of shrinkage, and the λ values were chosen by 10-time cross-validations using the 1-SE criteria. A formula was derived to generate the prediction model on the basis of variables selected from the LASSO Cox regression model, and weighted using the Cox regression coefficient. X-tile plots were used to generate the optimum cutoff point for the formula 37. A nomogram was constructed to determine the mortality risk among individuals. The discrimination of the prediction model was measured using the concordance index (C-index) and areas under the time-dependent receiver-operating characteristics (ROC) curves (AUCs), while the calibration of the prediction model was graphically explored using calibration plots. A bootstrap with 1000 re-samples was used for internal validation.

SAS 9.4 (SAS Institute Inc., Cary, NC, USA) and R 3.5.1 were used for the statistical analysis. The following R packages were used: “rms” to construct the nomogram, “glmnet” to conduct the LASSO, and “survival” and “timeROC” to determine the C-index and AUCs, respectively. A *P*-value of <0.05 was considered statistically significant.

## Results

### Treatment outcomes

Among 121 intermediate risk AML patients aged ≤65 years, seventy-four patients (74/121, 61.2%) achieved CR after one cycle of induction, and 23 patients (19.0%) after two cycles of induction. 46 patients (46/121, 38.1%) were treated with alloHSCT, and 75 patients (61.9%) received PR-CT. The 3-year OS and RFS were 48.3±5.1% and 36.5±5.0%, respectively. As expected, survival outcomes were in favor of transplantation arm. (OS: alloHSCT 61.2±8.0%, PR-CT 40.8±6.3%, *P*=0.044; RFS: alloHSCT 56.1±8.0%, PR-CT 26.5±5.7%,* P*=0.003; Supplementary Figures S2C-2D).

Forty-two patients (34.7%) relapsed. Among these patients, eight patients relapsed after alloHSCT. The 3-year CIR was significantly lower in patients who received alloHSCT when compared to patients treated with PR-CT (16.53±0.34% *vs.* 60.07±0.47%,* P*<0.001, Supplementary Figure S2E). NRM was slightly higher in the alloHSCT group than in the PR-CT group (24.27±0.48% *vs.* 9.86±0.15%,* P*=0.080; Supplementary Figure S2F).

### Mutation spectrum

**Figure 1:**
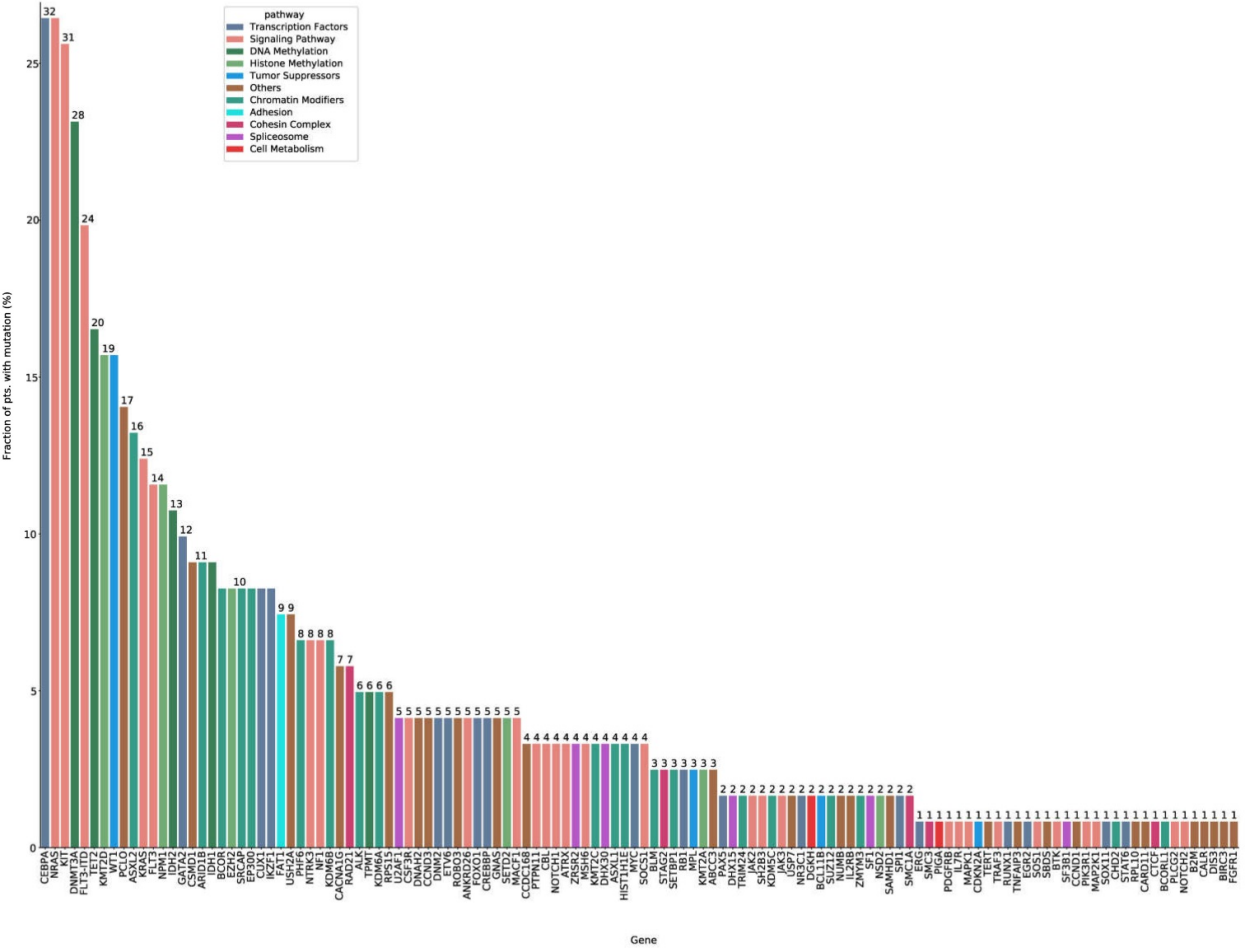
The mutation spectrum of intermediate risk AML patients (*n*=121). The frequency of mutations detected in ≥ 10 patients. The bars are color-coded in accordance with the common functional classification of pathways assigned to each mutated gene. *CEBPA* and *NRAS* mutation frequencies were the highest (*CEBPA*: *n*=32, 26.4%; *NRAS*: *n*=32, 26.4%), followed by those of *KIT* (*n*=31, 25.6%), *DNMT3A* (*n*=28, 23.1%), and *FLT-ITD* (*n*=24, 19.8%).

In total, 122 mutated genes were detected in 121 intermediate risk AML patients. Among these, 24 genes were mutated in more than 10 patients (Figure 1, Supplementary Table S3, Supplementary Table S4). *CEBPA* (26.4%) and *NRAS* (26.4%) mutations were the most common molecular events for the entire cohort, followed by *KIT* (25.6%), *DNMT3A* (23.1%) and *FLT3-ITD* (19.8%) mutations (Supplementary Table S3). The median number of mutated genes per patient was six (range: 0-11), and the median number of mutations was seven (range: 0-14). Median variant allele frequency (VAF) of these mutated genes was 0.42. The median VAFs of genes involved in signaling pathways was the lowest (0.16), suggesting that mutations in signaling pathways were acquired in the relatively late phase of AML pathogenesis (Supplementary Figure S3).

Significant co-occurrence was observed for *IKZF1* and *CEBPA*, *EZH2* and *CEBPA*, *GATA2* and *CEBPA*, *FLT3-ITD* and *DNMT3A*, *IDH1* and *DNMT3A*, *NPM1* and *DNMT3A*, *ASXL2* and *KIT*, *NPM1* and *FLT3-ITD* mutations (Supplementary Figure S4).

### Correlations between molecular profile and treatment outcomes

**Figure 2:**
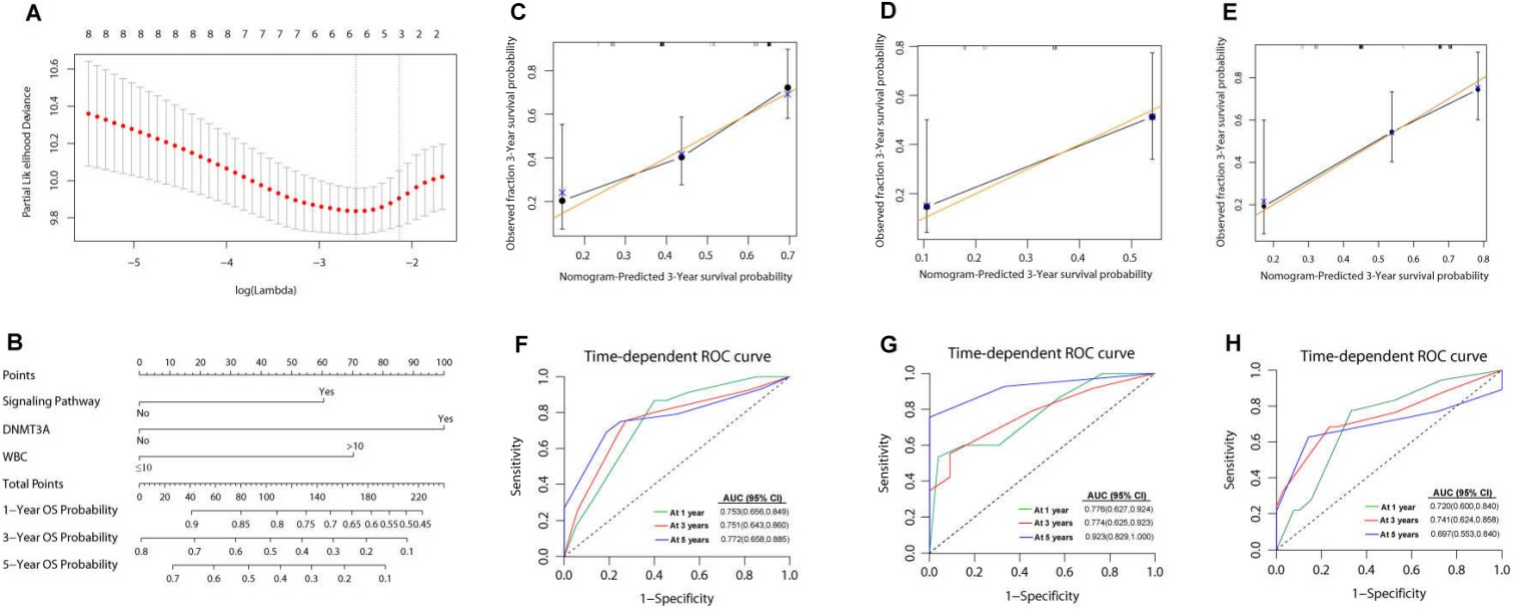
Nomogram for patients with intermediate risk AML. (A) Cross-validation for tuning parameter selection in the LASSO model. The solid vertical lines are binomial deviance±standard error (SE). Dotted vertical lines were drawn at the optimal values per the minimum criteria and 1-SE criteria. We plotted the partial likelihood deviance versus log (λ), where λ is the tuning parameter.Herein, a value of ln(λ)=-2.12 was selected through 10-time cross-validations via 1-SE criteria. (B) The nomogram based on data from 110 intermediate risk AML patients to predict individual prognosis. The calibration curves of an alternative nomogram to predict 3-year OS of intermediate risk AML patients (C, *n*=110), cohort 1 (D, *n*=41) and cohort 2 (E, *n*=99). The x-axis represents the predicted survival probability calculated using the nomogram, while the y-axis represents the actual survival probability for patients in the present study. The gold 45-degree line represents the ideal nomogram, while the black line represents the observed nomogram. The AUC values were 0.772-0.753 (F), 0.774-0.923 (G) and 0.697-0.741 (H) in the 110 intermediate risk AML patients, cohort1 and cohort 2, respectively.

Then we analyzed the impact of several variables on survival outcomes in 121 intermediate risk AML patients who were ≤ 65 years. In univariate analysis, an age of ≥55 years old at diagnosis, treatment modality (PR-CT), WBC count ≥ 10×10^9^/L at diagnosis, and *DNMT3A* mutation were associated with both reduced OS and an increased risk of relapse (Supplementary Table S5). To perform a multivariate analysis and establish a prognostic prediction model, the LASSO method was adopted for simultaneous shrinkage and variable selection. The optimal tuning parameter identified three variables: mutations in signaling pathway genes, *DNMT3A* mutation status, and WBC count at diagnosis, as independent factors for OS (Figure 2A, Supplementary Table S6).

Next, we evaluated the characteristics of patients with *DNMT3A* (DNMT3A^mut^) mutation in comparison with those with wild type *DNMT3A* (DNMT3A^wt^) (Supplementary Table S7). It was found that DNMT3A^mut^ patients were significantly older than DNMT3A^wt^ patients (median: 47 *vs.* 41 years, *P*=0.009), and more likely to have higher PLT counts at diagnosis (median: 91.5 *vs.* 30×10^9^/L, *P*=0.011). The 3-year OS and RFS for DNMT3A^mut^ patients was 24.0±9.3% and 21.2±9.3%, respectively, and 54.8±5.7% and 46.5±5.9% for DNMT3A^wt^ patients (*P*=0.001 and *P*=0.009, respectively; Supplementary Figures S5A-5B). The survival for intermediate risk patients with DNMT3A^mut^ (*n*=28) was as poor as that of adverse risk AML patients with the same age group (*n*=65; 3-year OS: 24.0±9.3% *vs.* 33.3±6.9%, *P*=0.468; RFS: 20.2±9.3% *vs.* 39.1±7.4%, *P*=0.247; Supplementary Figures S5C-5D).

### Development of a prediction model combining clinical features and mutation profiles

A nomogram was developed with the risk factors for OS from the multivariate analysis. The risk score was calculated using the following equation: 0.6749 × signaling pathway + 1.1147 ×* DNMT3A* + 0.7829 × WBC; where signaling pathway and *DNMT3A* equals 1 for the presence and 0 for the absence of a respective feature, and WBC equals 1 if the WBC count was higher than 10×10^9^/L (Figure 2B). The genes in signaling pathways included *NRAS*, *KIT*, *FLT3-ITD*, *FLT3*, *KRAS*, *NF1*, *NTRK3*, *MACF1*, *ANKRD26*, *NOTCH1*, *CSF3R*, *PTPN11*, *CBL*, *MSH6*, *ATRX*, *SOCS1*, *JAK3*, *JAK2*, *SH2B3*, *MAP2K1*, *PIK3R1*, *PLCG2*, *ATM*, *SOS1*, *TRAF3*, *IL7R*, *MAPK1*, *BTK*,* NOTCH2* and *PDGFRB*. The presence of any mutations in these genes equals 1. The predictive accuracy for OS and RFS calculated using the C-index was 0.703 (95% CI: 0.643-0.763) and 0.681 (95% CI: 0.620-0.741), respectively. In the internal validation, the corrected C-index of OS was 0.697 after bootstraps resampling. Similarly, in the validation calculations, the C-index for OS in cohort 1 was 0.708 (95% CI: 0.620-0.797). In cohort 2, the Cindex of OS and RFS was 0.708 (95% CI: 0.638-0.779) and 0.680 (95%CI: 0.617-0.744), respectively.

The calibration curves of the alternative nomogram to predict the 3-year OS presented in Figures 2C-2E suggested a good fit for the observed nomogram, when compared with the ideal nomogram. The panel displayed an AUC value of 0.753 (95% CI: 0.656-0.849) at 1-year OS, 0.751 (95% CI: 0.643-0.860) at 3-year OS, and 0.772 (95% CI: 0.658-0.885) at 5-year OS (Figure 2F). For validation sets, the panel had high AUC values at these timepoints (cohort 1: 0.776 [95% CI: 0.627-0.924], 0.774 [95% CI: 0.625-0.923], and 0.923 [95% CI: 0.829-1.000], respectively; cohort 2: 0.720 [95% CI: 0.600-0.840], 0.741 [95%CI: 0.624-0.858], and 0.697 [95%CI: 0.553-0.840]; Figure 2G-2H).

**Figure 3:**
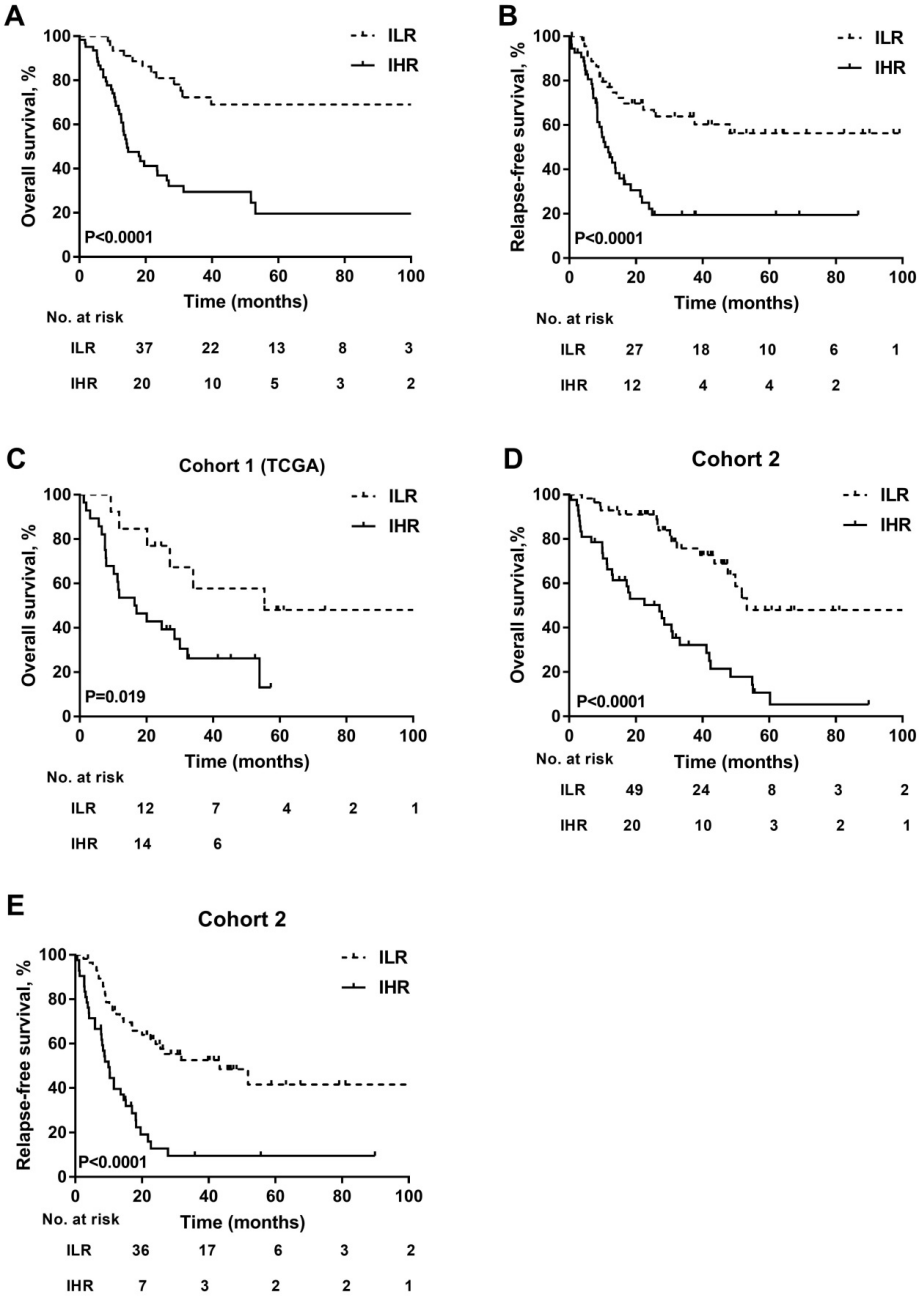
Kaplan-Meier curves for OS and RFS for patients withwith different risk score. (A) OS; (B) RFS. The results were validated in cohort 1 (C) and cohort 2 (D-E).

The cut-off point of these risk scores was 1.00. Accordingly, the patients were divided into two subgroups: intermediated-low risk (ILR) group (risk score <1, n=48) and intermediated-high risk (IHR) group (risk score ≥1, n=62). The comparison of clinical characteristics between derivation and validation groups is presented in Table 2. The 3-year OS and RFS in patients with ILR AML were significantly better than those of patients with IHR AML (3-year OS: 72.3±7.2% *vs.* 29.5±6.6%, *P*<0.0001, Figure 3A; 3-year RFS: 63.9±7.6% *vs.* 19.4±6.3%, *P*<0.0001; Figure 3B). Similar results were obtained in the validation of cohort 1 (ILR *vs.* IHR: 3-year OS, 57.7±14.7% *vs.* 26.2±8.7%, *P*=0.019; Figure 3C) and cohort 2 (ILR vs. IHR: 3-year OS, 75.7±6.6% *vs.* 32.2±7.8%, *P*<0.0001; 3-year RFS: 52.5±7.1% *vs.* 9.6±5.1%, *P*< 0.0001; Figure 3D-3E). A risk score of ≥1 indicated a risk of 4.43 (95% CI: 2.30-8.52, *P*<0.0001) for death and 3.10 (95% CI: 1.72-5.59, *P*<0.0001) for relapse. As shown in Supplementary Figure S6, the prognosis of patients with a risk score of ≥1 was as poor as those of adverse risk AML (*n*=65, 3-year OS: 29.5±6.6% *vs.* 33.3±6.9%, *P*=0.794; 3-year RFS: 19.4±6.3% *vs.* 39.1±7.4, *P*=0.294).

**Figure 4:**
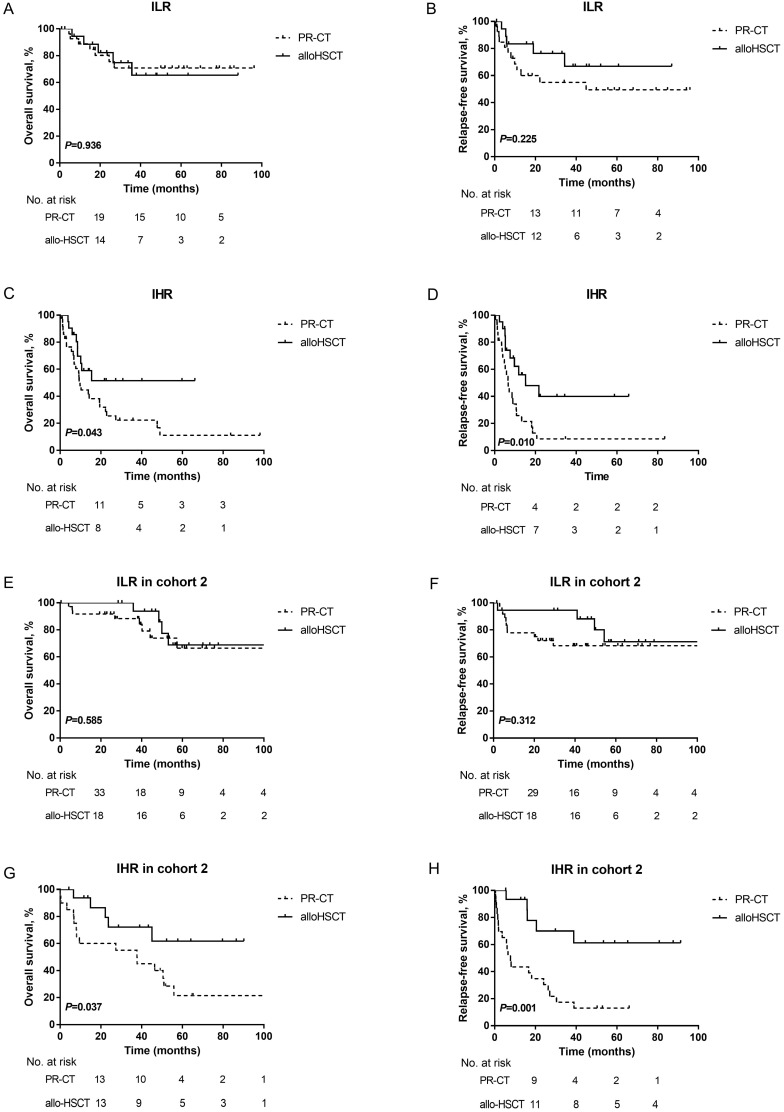
Kaplan-Meier curves for OS and RFS. (A) OS and (B) RFS according to different PRT modalites in ILR group of the training cohort. (C) OS and (D) RFS according to different PRT modalites in IHR group of the training cohort. A landmark was set at 4 months for OS and 3 months for RFS in the training cohort. (E) OS and (F) RFS according to different PRT modalites in ILR group of cohort 2. (G) OS and (H) RFS according to different PRT modalites in IHR group of cohort 2. A landmark was set at 3 months for OS and 2 months for RFS in cohort 2.

Furthermore, the prognosis of patients in the two subgroups with different PRT was evaluated by landmark analysis. PRT modalities did not influence the survival of ILR patients at the landmark date (3-year OS: PR-CT: 70.8±9.4% *vs.* alloHSCT: 65.4±13.1%, *P*=0.936; 3-year RFS: PR-CT: 55.0±10.2% *vs.* alloHSCT: 66.8±12.8%, *P*=0.225; Figures 4A-4B). However, patients in the IHR group who underwent alloHSCT had a survival advantage over those treated with PR-CT (3-year OS: 51.6±12.0% *vs.* 22.3±7.4%, *P*=0.043; 3-year RFS: 40.0±12.8% *vs.* 8.6±5.8%, *P*= 0.010; Figures 4C-4D). Due to the unavailable data in cohort 1, the landmark analysis was only performed in cohort 2. Consistent results were observed (Figure 4 E-4H).

### Comparison of prediction model

**Figure 5:**
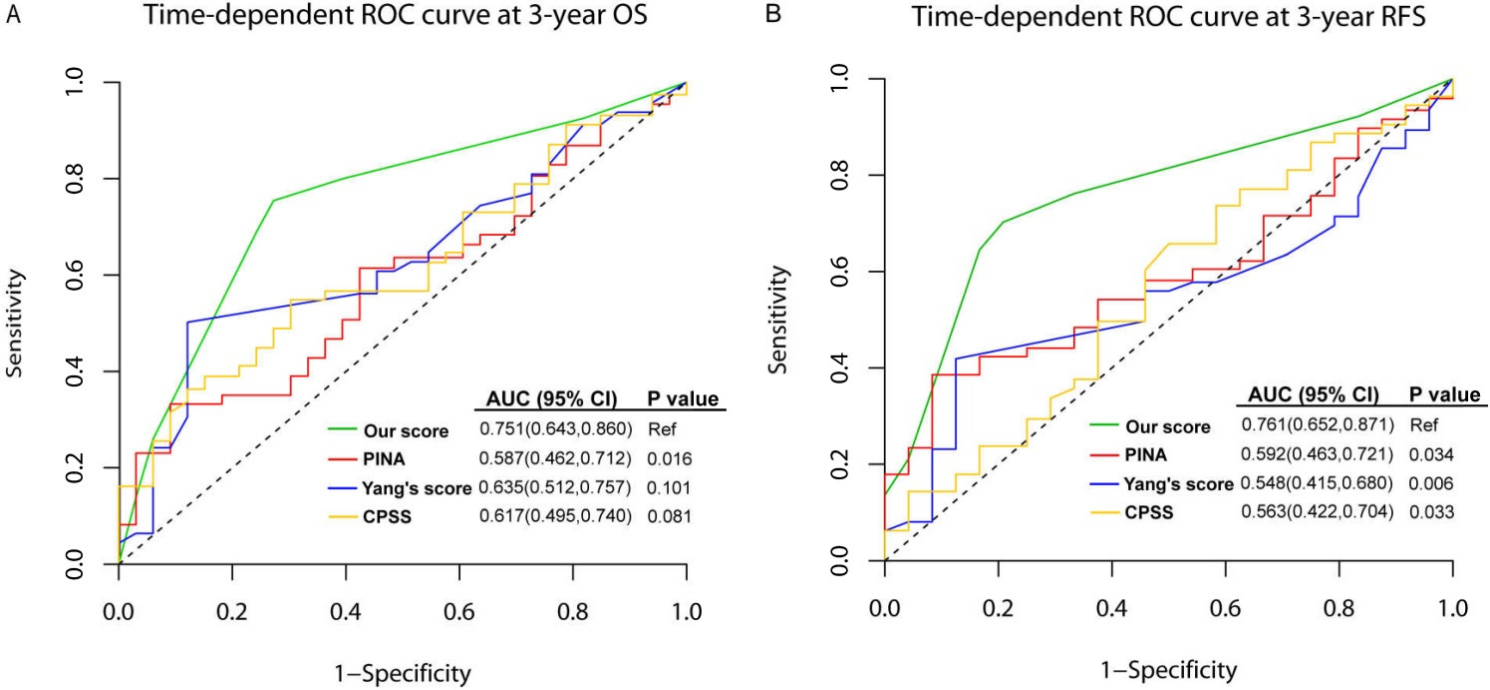
The comparison between the present prediction model for intermediate-risk AML and published models in the training set. ROCs of the prediction model for intermediate-risk AML, PINA score, Yang's score system, and CPSS score. (A) time-dependent ROC curve at 3-year OS; (B) time-dependent ROC curve at 3-year RFS.

To assess the quality and value of the present prediction model in intermediate-risk AML, we compared the AUCs of this model with other published prediction models in training set, which were PINA score (including *NPM1*, *FLT3-ITD*, *CEBPA*, WBC, age and ECOG) 38, Yang' score system [including age, hematopoietic cell transplantation-comorbidity index (HCT-CI), WBC, Hb,* CEBPA*, *DNMT3A*, *FLT3-ITD*, *NPM1*, ELN risk status] 39, and CPSS score (including age, FAB, LDH, ECOG, cytogenetics, favorable or unfavorable gene mutations, chromatin-spliceosome) 40. As shown in Figure 5, the AUC of the present model was higher than other three models in predicting 3-year OS, although only the difference between our model and PINA score was significant [0.751 (95%CI: 0.643-0.860) *vs.* 0.587 (95%CI: 0.462-0.712), *P*=0.016]. For 3-year RFS, our model showed statistically higher AUC than others [our score: 0.761 (95%CI: 0.652-0.871), PINA score: 0.592 (0.463-0.721), Yang's score: 0.548 (0.415-0.680), CPSS score: 0.563 (0.422-0.704), all *P* values <0.05].

## Discussion

Most studies on the mutation landscape of AML have focused on general AML population, AML with normal cytogenetics, or the comparison of mutation spectrums between young and old patients 26, 41-44. In the present study, intermediate risk patients were recruited in accordance with the 2017 ELN criteria with strict age limits to increase the homogeneity of the population. It was found that 99.2% (120/121) of AML patients had at least one mutation detected by targeted NGS. Consistent with previous reports 22, 45, 46, only 24 genes were mutated in > 10% patients, with *CEBPA* and *NRAS* mutations being the most common (26.4%). In a recent study reported by Wang *et al.*, the frequency of *CEBPA* mutation in intermediate risk AML was similar to our observations (28.4%) 46. Previous studies reported a specific association between *CEBPA* and *GATA2* transcription factor mutations was observed in 35%-39% of cases 42, 47. *GATA2* mutation was also detected in 24.2% of patients with *CEBPA* mutations in our cohort. The frequency of *NPM1* mutations in the present cohort (11%) was lower than that reported in other studies 16, 46, 48. Mutated *NPM1* without *FLT3-ITD* or with* FLT3-ITD^low^* is categorized as favorable risk in 2017 ELN classification, thus decreasing the *NPM1* mutation frequency in intermediate-risk group.

The prognostic value of* DNMT3A* mutations in AML has been previously reported 26, 48, 49. Young patients with *DNMT3A* mutation were predisposed to have a lower CR rate after induction therapy 48. Herein, there were not any strong connections between *DNMT3A* mutation and CR rate. In a large cohort of AML patients reported by Ley *et al.*, 24 *DNMT3A* mutation was an independent predictor for inferior survival in patients with intermediate risk cytogenetics. *DNMT3A* R882H mutation exerts a dramatic effect on transcriptional regulation 50. In a *DNMT3A* R882H mutant model, a significant increase in PLT count were observed along with *Mpl* upregulation. The protein encoded by *Mpl*, regulates both megakaryocytic progenitors and other myeloid progenitor cells 51. Consistent with our previous study, DNMT3A^mut^ patients were predisposed to higher PLT counts (≥40×10^9^/L) at diagnosis, and higher PLT counts were associated with poor survival outcome in intermediate risk group 52. On the other hand, concomitant mutation of *NPM1* and *FLT-ITD with DNMT3A* was observed in our cohort as frequently found in other independent cohorts 53, 54. Guryanova et al. reported the interactions among the three mutated genes 55. It was recently reported that simultaneous mutations in the three genes was highly associated with the expression of GPR56, a leukemia stem cell marker 56. These evidences further demonstrated that the three-gene mutated AML was with very poor outcome.

Long-term survival widely varied among intermediate risk AML patients. The ELN stratified cytogenetically normal AML patients into two risk groups with the mutation profiles of *NPM1*, *FLT3-ITD* and *CEBPA* 11. The reference models with which our prediction model should be compared are the studies from Patel and Hou's 22, 27. They developed 18- and 8-gene panels, respectively, to reclassify cytogenetic intermediate-risk AML into favorable, intermediate and unfavorable subgroups. These studies, including Wakita's 57, potentially reduced the proportion of intermediate-risk AML patients, however, these risk profiles were not correlated with treatment modalities. The models identified patients with high risk, but not those for whom alloHSCT significantly prolonged the RFS. We did not observe distinct benefits of alloHSCT for low risk patients within our predictive model. Nevertheless, we demonstrated that alloHSCT would benefit for intermediate risk patients with high risk score, which suggested that high-risk patients benefit substantially from alloHSCT. Tsai et al. recently incorporated long non-coding RNA (IncRNA) expression profiles to refine the 2017 ELN risk classification 58. The IncRNA scoring system helped dichotomize patients into two groups. However, their intermediate-risk group was a very small cohort (n=29). Additionally, analysis of IncRNA expression was costly and not widely applicable in clinical settings, thus restricting its widespread application. To our knowledge, our study is the first to comprehensively investigate genetic alterations in 2017 ELN intermediate risk AML. Based on the prediction model we proposed, intermediate risk AML patients who can truly benefit from alloHSCT over chemotherapy can be easily and precisely identified upon diagnosis with a higher efficiency as shown in Figure 5.

The risk profile was derived from the retrospective study is the potential limitation to our work. Furthermore, the sizes of the derivation and validation cohorts are relatively small. Further prospective studies with more patients recruited are needed to verify this point.

In conclusion, we described the genetic mutation landscape of young patients with intermediate risk AML. The integration of clinical and molecular profiles would allow evaluation of the possible benefits of alloHSCT for IHR patients who can be rapidly recognized by at diagnosis.

## Supplementary Material

Supplementary figures and tables.

## Figures and Tables

**Table 1:** Clincal features of intermediate risk AML patients ≤65 years old.

Parameters	Number of patients
Total cohort	121
Males, *n* (%)	70 (57.9%)
Females, *n* (%)	51 (42.1%)
Median age(range), years	44 (14-65)
Laboratory parameters	
WBC count, median (range) ×10^9^/L	11.6 (0.5-210.0)
Platelet count, median (range) ×10^9^/L	31.0 (4.0-561.0)
Hemoglobin count, median (range) ×g/L	87.5 (42.0-204.0)
BM blast, median (range) %	64.5 (21.0-98.1)
LDH, median (range) U/L	260.0 (61.0-2567.0)
ECOG Performance Status at Diagnosis, *n* (%)	
≤1	77 (63.6)
2	38 (31.4)
3	6 (5.0)
Normal karyotype	63 (52.0)
Other	58 (39.7)
CR reached after	
Cycle1 (early CR)	74 (61.2)
Cycle2 (late CR)	23 (19.0)
Other	24 (19.8)
Hematopoietic stem cell transplantation, *n* (%)	
No	75 (61.9)
Yes	46 (38.1)
Relapse, *n* (%)	
No	79 (65.3)
Yes	42 (34.7)
Death, *n* (%)	
No	65 (53.7)
Yes	56 (46.3)
Treatment, *n* (%)	
DA	101 (83.5)
D-CAG	12 (9.9)
Other	8 (6.6)
3-year OS, %	48.3±5.1
3-year RFS, %	36.5±5.0
Follow-up, median (range) months	35.2 (1.1-102.4)

Abbreviations: WBC, white blood cell count; BM, bone marrow; LDH, lactate dehydrogenase, MRD, minimal residual disease before cycle1, cycle2 or before post remission chemotherapy; ECOG, Eastern Cooperative Oncology Group, AML, acute myeloid leukemia; FAB, French-American British classification; NOS, non-specific type; CR, complete remission; DA, daunorubicin +cytarabine; D-CAG, decitabine combined with low-dose arabinosylcytosine (Ara-c), aclarubicin and granulocyte colony-stimulating factor (G-CSF); OS, overall survival (with event death whatever the cause); RFS, relapse-free survival (with event death in first CR or relapse).

**Table 2:** Basic characteristics of patients according to the proposed risk score in intermediate risk AML patients (*n*=110), the cohort 1(*n*=41) and cohort 2 (n=99).

Variables	ELN defined intermediate risk AML (*n*=110)			Cohort 1 (TCGA) (*n*=41)			Cohort 2 (n=99)		
	ILR (risk score < 1) (*n*=48)	IHR (risk score ≥ 1) (*n*=62)	*P*	ILR (*n*=13)	IHR (*n*=28)	*P*	ILR (*n*=56)	IHR (*n*=43)	*P*
**Score median (range)**	0.67 (0,0.67)	1.46 (1.11,2.57)	**<0.001**	0.78 (0, 78)	1.79 (1.46,2.57)	**<0.001**	0.67 (0, 0.78)	1.46 (1.11, 2.57)	**<0.001**
Gender, males, *n* (%)	31 (64.58)	34 (54.84)	0.303	8 (61.54)	11 (39.29)	0.184	32 (57.14)	23 (53.49)	0.717
Females, *n* (%)	17 (35.42)	28 (45.16)		5 (38.46)	17 (60.71)		24 (42.86)	20 (46.51)	
Age, median (range), years	44 (16, 61)	44.5 (14, 65)	0.290	51 (22,63)	56.5 (25,65)	0.227	44 (19,64)	43 (15,65)	0.432
Laboratory parameters									
**WBC count, median (range)×10^9^/L**	4.35 (0.54, 207.73)	25.17 (0.70,210)	**<0.001**	12.1 (0.6,50.3)	47 (1.20,202.70)	**0.001**	4.94 (1.10,202.13)	53.80 (3.60,405.13)	**<0.001**
PLT, median (range) ×10^9^/L	30 (4, 268)	51.5 (5, 561)	0.139	NA	NA	NA	NA	NA	
Hb, median (g/L, range)	86 (47, 148)	88.5 (2, 204)	0.519	NA	NA	NA	NA	NA	
BM blast, median (range) %	63 (22, 96.5)	67 (21,98)	0.734	61 (34,95)	81.5 (39,100)	**0.025**	57 (21.5,94.2)	73.6 (20,94.4)	0.037
**LDH, median (range) U/L**	226 (61, 1370)	353 (97, 2567)	**0.003**	NA	NA	NA	NA	NA	
ECOG at diagnosis, *n* (%)			0.595	NA	NA	NA	NA	NA	NA
≤1	32 (66.67)	38 (61.29)		NA	NA	NA	NA	NA	NA
2	15 (31.25)	20 (32.26)		NA	NA	NA	NA	NA	NA
3	1 (2.08)	4 (6.45)		NA	NA	NA	NA	NA	NA
***DNMT3A* mutation**			**<0.001**			**<0.001**			**<0.001**
No	48 (100)	37 (59.68)		13 (100)	11 (39.29)		56 (100)	25 (58.14)	
Yes	0 (0)	25 (40.32)		0 (0)	17 (60.71)		0 (0)	18 (41.86)	
**Mutations in signaling Pathway**			**<0.001**						
No	20 (41.67)	5 (8.06)		12 (92.31)	4 (14.29)	**<0.001**	39 (69.64)	3 (6.98)	**<0.001**
Yes	28 (58.33)	57 (91.94)		1 (7.69)	24 (85.71)		17 (30.36)	40 (93.02)	
Cytogenetics, *n* (%)			**0.016**			0.790			**0.022**
Normal karyotype	32 (66.67)	27 (43.55)		8 (61.54)	16 (57.14)		29 (51.79)	32(74.42)	
Other	16 (33.33)	35 (56.45)		5 (38.46)	12 (39.29)		27 (48.21)	11 (25.58)	
CR reached after			**0.017**			NA			0.246
Cycle1 (early CR)	31 (64.58)	37 (59.68)		NA	NA		34 (60.71)	22 (51.16)	
Cycle2 (late CR)	13 (27.09)	8 (12.90)		NA	NA		15 (26.79)	10 (23.26)	
Other	4 (8.33)	17 (27.42)		NA	NA		7 (12.50)	11 (25.58)	
alloHSCT, *n* (%)			0.597			0.790			
No	31 (64.58)	43 (69.35)		5 (38.46)	12 (42.86)		38 (67.86)	26 (60.47)	0.446
Yes	17 (35.42)	19 (30.65)		8 (61.54)	16 (57.14)		18 (32.14)	17 (39.53)	
Relapse, *n* (%)			0.484			NA			**0.036**
No	34 (70.83)	40 (64.52)		NA	NA		38 (67.86)	37 (86.05)	
Yes	14 (29.17)	22 (35.48)		NA	NA		18 (32.14)	6 (13.95)	
**3-year OS (%)**	72.3±7.2	29.5±6.6	**<0.0001**	57.7±14.7	26.2±8.7	**0.019**	75.7±6.6	32.2±7.8	**<0.0001**
**3-year RFS (%)**	63.9±7.6	19.4±6.3	**<0.0001**	NA	NA	NA	52.5±7.1	9.6±5.1	**<0.0001**

Abbreviations: WBC, white blood cells; PLT, platelet; Hb, hemoglobin; BM, bone marrow; LDH, lactate dehydrogenase; CR, complete remission; OS, overall survival; RFS, relapse free survival; NA, not available.

## Abbreviations

AML: acute myeloid leukemia
ILR: intermediate low risk
IHR: intermediate high risk
OS: overall survival
RFS: relapse-free survival
C-index: Concordance index
alloHSCT: allogeneic hematopoietic stem cell transplantation
NGS: next generation sequencing
ELN: European LeukemiaNet
PRT: post-remission therapy
NRM: non-relapse mortality
ECOG: Eastern Cooperative Oncology Group
TCGA: The Cancer Genome Atlas
CR: complete remission
PLT: platelet
CIR: cumulative incidence of relapse
LASSO: the Least Absolute Shrinkage and Selector Operation
AUC: areas under the time-dependent receiver-operating characteristics curves
HCT-CI: hematopoietic cell transplantation-comorbidity index
IncRNA: long non-coding RNA
