# Supplementary material for: A clinical prediction model identifies a subgroup with inferior survival within intermediate risk acute myeloid leukemia
Source: J Cancer. 2021 Jun 11;12(16):4912–23. doi: 10.7150/jca.57231 (PMC8247394; doi:10.7150/jca.57231)
Supplement: Supplementary file 1 — Supplementary figures and tables. [file jcav12p4912s1.pdf]

Supplementary Table S1. Baseline characteristics and treatment details for the training and test cohorts.

	ELN defined intermediate risk AML age ≤65 years: training set	Cohort 1 (TCGA)	Cohort 2	*P*
	n= 110	n= 41	n= 99	
Gender, *n* (%)				0.374
Males	65 (59.1)	19 (46.3)	55 (55.6)	
Females	45 (40.9)	22 (53.7)	44 (44.4)	
Age, median (range), years	44 (14,65)	54 (22,65)	43 (15,65)	**0.032**
Laboratory parameters				
WBC count, median (range) ×109/L	11.66 (0.54,210)	33.5 (0.6,202.7)	13.89 (1.1,405.13)	0.254
PLT, median (range) ×109/L	31 (4,561)	NA	NA	NA
Hb, median (range) g/L	87.5 (2,204）	NA	NA	NA
BM blast, median (range) %	65.5 (21,98)	67 (34,100)	66 (20,94.4)	0.058
LDH, median (range) U/L	253 (61,2567)	NA	NA	NA
ECOG at Diagnosis, *n* (%)				NA
≤1	70 (63.6)	NA	NA	
2	35 (31.8)	NA	NA	
3	5 (4.6)	NA	NA	
DNMT3A mutation, *n* (%)				**0.012**
No	85 (77.3)	24 (58.5)	81 (81.8)	
Yes	25 (22.7)	17 (41.5)	18 (18.2)	
Mutations in signaling Pathway, *n* (%)				**0.007**
No	25 (22.7)	16 (39.0)	42 (42.4)	
Yes	85 (77.3)	25 (61.0)	57 (57.6)	
Cytogenetics, *n* (%)				0.503
Normal karyotype	59 (53.6)	24 (58.5)	61 (61.6)	
Other	51 (46.4)	17 (41.5)	38 (38.4)	
CR reached after, *n* (%)				0.347[a]
Cycle1 (early CR)	68 (61.8)	NA	56 (56.6)	
Cycle2 (late CR)	21 (19.1)	NA	24 (24.2)	
Median time from diagnosis to CR (range, d)	59 (20, 215)	NA	73 (43, 162)	**<0.001[a]**
Median time from CR to alloHSCT (range, d)	96 (79, 445)	NA	67 (52-269)	0.449[a]
Median time from diagnosis to alloHSCT (range, d)	123 (105, 660)	NA	102 (89-573)	0.631[a]
alloHSCT, *n* (%)				**0.012**
No	70 (63.6)	17 (41.5)	67 (67.7)	
Yes	40 (36.4)	24 (58.5)	32 (32.2)	

WBC, white blood cells; PLT, platelet; Hb, hemoglobin; BM, bone marrow; LDH, lactate dehydrogenase; CR, complete remission; OS, overall survival; RFS, relapse free survival; MB, mutation burden; NA, not available; a: training set versus Cohort2.

Supplementary Table S2. 210-gene panel for AML

ABCC3	CCND1	DNM2	IDH1	MYC	PRPF8	SOX11
ABL1	CCND3	DNMT3A	IDH2	MYD88	PRPS1	SPI1
AKT2	CD28	DOK5	IKZF1	NF1	PTEN	SRCAP
AKT3	CD58	EGR2	IL2RB	NF2	PTPN11	SRP72
ALK	CD79A	ELANE	IL7R	NFKBIA	RAD21	SRSF2
AMER1	CD79B	EP300	IRF4	NFKBIE	RB1	STAG1
ANKRD26	CDKN1B	EPOR	ITPA	NOTCH1	RHOA	STAG2
ARID1B	CDKN2A	ERG	JAK1	NOTCH2	RIT1	STAT3
ARID2	CEBPA	ETNK1	JAK2	NPM1	ROBO1	STAT5A
ASXL1	CECR2	ETV6	JAK3	NR3C1	ROBO2	STAT5B
ASXL2	CEP72	EVI1	KDM5C	NRAS	ROBO3	STAT6
ATM	CHD2	EZH2	KDM6A	NSD2	RPL10	SUZ12
ATRX	CHD8	FAM46C	KDM6B	NT5C2	RPS15	TCF3
B2M	CREBBP	FAT1	KIT	NTRK1	RRM1	TERC
BCL11B	CRLF2	FBXW7	KMT2A	NTRK3	RRM2B	TERT
BCL2	CSF3R	FGFR1	KMT2C	NUDT15	RUNX1	TET2
BCL6	CSMD1	FLT3	KMT2D	NUMB	SAMHD1	TNFAIP3
BCOR	CTCF	FOXO1	KRAS	PAX5	SBDS	TNFRSF14
BCORL1	CTLA4	GART	MACF1	PCLO	SETBP1	TP53
BIRC3	CUX1	GATA1	MAP2K1	PDGFRA	SETD2	TPMT
BLM	CXCR4	GATA2	MAP2K4	PDGFRB	SF1	TRAF3
BMP7	CYP3A4	GATA3	MAP3K7	PHF6	SF3B1	TRIM24
BRAF	CYP3A5	GFI1	MAPK1	PIGA	SH2B3	U2AF1
BTK	DDX41	GNA13	MED12	PIK3CA	SLCO1B1	USH2A
CACNA1E	DGKH	GNAS	MEF2B	PIK3R1	SMAD4	USP7
CACNA1G	DHX15	GSTM1	MLH1	PLCG2	SMC1A	VEGFC
CALR	DHX30	GSTP1	MPL	PNPLA3	SMC3	WT1
CARD11	DIS3	HAX1	MSH6	POT1	SOCS1	XPO1
CBL	DKC1	HIST1H1E	MTHFR	PPM1D	SOD2	ZMYM3
CCDC168	DNAH2	ID3	MTRR	PRKDC	SOS1	ZRSR2

Supplementary Table S3. The gene mutations found in ≥10 intermediate risk AML patients (n = 121).

Genes	Number of Patients, n (%)
CEBPA	32(26.4%)
NRAS	32(26.4%)
KIT	31(25.6%)
DNMT3A	28(23.1%)
FLT3-ITD	24(19.8%)
TET2	20(16.5%)
WT1	19(15.7%)
KMT2D	19(15.7%)
PCLO	17(14.0%)
ASXL2	16(13.2%)
KRAS	15(12.4%)
FLT3	14(11.6%)
NPM1	14(11.6%)
IDH2	13(10.7%)
GATA2	12(9.9%)
IDH1	11(9.1%)
ARID1B	11(9.1%)
CSMD1	11(9.1%)
EP300	10(8.3%)
SRCAP	10(8.3%)
CUX1	10(8.3%)
EZH2	10(8.3%)
BCOR	10(8.3%)
IKZF1	10(8.3%)

Supplementary Table S4. Somatic non-silent mutations identified in 120 patients* subject to targeted sequencing

Patient ID	Chromosome	Start	Base change	End	Variant allele frequency	Mutation type	Transcription ID	Gene	Amino acid change
P1	chr11	32414280	c.1270_1271insTG	32414280	0.4768	frameshift insertion	NM_024426	WT1	p.D424fs
P1	chr20	57429696	c.1376_1377insTGACGCCCCAGCCGATCCCGACTCCGGGGCGGCCCG	57429696	0.543	nonframeshift insertion	NM_080425	GNAS	p.P459delinsPDAPADPDSGAAR
P1	chrX	76937102	c.3646A>G	76937102	0.494	nonsynonymous SNV	NM_000489	ATRX	p.I1216V
P1	chr8	2830663	c.8899T>A	2830663	0.4973	nonsynonymous SNV	NM_033225	CSMD1	p.S2967T
P1	chr12	25398284	c.35G>A	25398284	0.4196	nonsynonymous SNV	NM_004985	KRAS	p.G12D
P1	chr15	91347516	c.3678C>A	91347516	0.4964	stopgain	NM_000057	BLM	p.C1226X
P2	chr4	106156365	c.1266delA	106156365	0.4729	frameshift deletion	NM_001127208	TET2	p.G422fs
P2	chr4	106158426	c.3327_3328del	106158427	0.4801	frameshift deletion	NM_001127208	TET2	p.S1109fs
P2	chr5	170837545	c.859_860insTCTG	170837545	0.4856	frameshift insertion	NM_002520	NPM1	p.L287fs
P2	chrX	39933478	c.1121A>G	39933478	0.0088	nonsynonymous SNV	NM_001123383	BCOR	p.K374R
P2	chr22	41525969	c.1244T>C	41525969	0.0067	nonsynonymous SNV	NM_001429	EP300	p.L415P
P2	chr4	1920208	c.1268C>T	1920208	0.4712	nonsynonymous SNV	NM_133330	NSD2	p.P423L
P2	chr7	82764227	c.2639G>A	82764227	0.5098	nonsynonymous SNV	NM_033026	PCLO	p.G880D
P2	chr12	112888197	c.213T>A	112888197	0.4978	nonsynonymous SNV	NM_002834	PTPN11	p.F71L
P2	chr2	25463296	c.2196dupT	25463296	0.4876	stopgain	NM_022552	DNMT3A	p.E733_F734delinsX
P3	chr19	33793113	c.196_208del	33793125	0.4402	frameshift deletion	NM_004364	CEBPA	p.A66fs
P3	chr12	49444946	c.2520_2522delinsGCA	49444944	0.0064	frameshift insertion	NM_003482	KMT2D	p.C841H
P3	chr11	32414262	c.1288dupC	32414262	0.0167	frameshift insertion	NM_024426	WT1	p.R430fs
P3	chr11	32450159	c.652_653insTACA	32450159	0.3448	frameshift insertion	NM_024426	WT1	p.S218fs
P3	chr19	33792431	c.890G>C	33792431	0.4775	nonsynonymous SNV	NM_004364	CEBPA	p.R297P
P3	chr4	187557941	c.3770G>A	187557941	0.4963	nonsynonymous SNV	NM_005245	FAT1	p.R1257Q
P3	chr7	50450389	c.573C>A	50450389	0.4569	nonsynonymous SNV	NM_006060	IKZF1	p.H191Q
P3	chr4	55593613	c.1679T>A	55593613	0.0056	nonsynonymous SNV	NM_000222	KIT	p.V560D
P3	chr4	55599321	c.2447A>T	55599321	0.281	nonsynonymous SNV	NM_000222	KIT	p.D816V
P3	chr1	115258748	c.34G>A	115258748	0.0359	nonsynonymous SNV	NM_002524	NRAS	p.G12S
P3	chr4	106180899	c.3927T>G	106180899	0.4711	nonsynonymous SNV	NM_001127208	TET2	p.F1309L
P3	chr11	32414238	c.1313T>C	32414238	0.0655	nonsynonymous SNV	NM_024426	WT1	p.L438P
P3	chr11	32456424	c.468C>A	32456424	0.0092	stopgain	NM_024426	WT1	p.C156X
P4	chr7	82764430	c.2436delA	82764430	0.0097	frameshift deletion	NM_033026	PCLO	p.K812fs
P4	chr13	28592642	c.2503G>T	28592642	0.012	nonsynonymous SNV	NM_004119	FLT3	p.D835Y
P4	chr4	55599320	c.2446G>T	55599320	0.424	nonsynonymous SNV	NM_000222	KIT	p.D816Y
P4	chr21	36164605	c.1270T>G	36164605	0.1663	nonsynonymous SNV	NM_001754	RUNX1	p.S424A
P5	chr5	170837545	c.859_860insTCTG	170837545	0.4671	frameshift insertion	NM_002520	NPM1	p.L287fs
P5	chr13	28608280	c.1775_1776insGGTGACCGGCTCCTCAGATAATGAGTACTTCTACGT	28608280	0.3149	nonframeshift insertion	NM_004119	FLT3-ITD	p.V592delinsVVTGSSDNEYFYV
P5	chr8	3889471	c.566C>G	3889471	0.5	nonsynonymous SNV	NM_033225	CSMD1	p.P189R
P5	chr3	47887236	c.986T>C	47887236	0.4978	nonsynonymous SNV	NM_138615	DHX30	p.M329T
P5	chr17	7705010	c.8814C>A	7705010	0.4843	nonsynonymous SNV	NM_020877	DNAH2	p.D2938E
P5	chr19	10828997	c.79T>G	10828997	0.5088	nonsynonymous SNV	NM_001005361	DNM2	p.C27G
P5	chr2	25457243	c.2644C>T	25457243	0.4831	nonsynonymous SNV	NM_022552	DNMT3A	p.R882C
P5	chr2	209113113	c.394C>G	209113113	0.4388	nonsynonymous SNV	NM_001282386	IDH1	p.R132G

P5	chr4	1978230	c.3650A>G	1978230	0.0056	nonsynonymous SNV	NM_133330	NSD2	p.K1217R
P6	chr13	41134889	c.739G>C	41134889	0.5006	nonsynonymous SNV	NM_002015	FOXO1	p.G247R
P6	chr6	26156833	c.215A>G	26156833	0.5167	nonsynonymous SNV	NM_005321	HIST1H1E	p.D72G
P6	chr6	18130918	c.719A>G	18130918	0.5369	nonsynonymous SNV	NM_000367	TPMT	p.Y240C
P7	chrX	123184981	c.1029_1032delinsGGCCTAGG	123184981	0.8969	frameshift insertion	NM_001042749	STAG2	p.V343fs
P7	chrX	39932819	c.1780G>A	39932819	1	nonsynonymous SNV	NM_001123383	BCOR	p.V594I
P7	chr7	50459526	c.815C>T	50459526	0.0557	nonsynonymous SNV	NM_006060	IKZF1	p.A272V
P7	chr1	115258747	c.35G>A	115258747	0.3127	nonsynonymous SNV	NM_002524	NRAS	p.G12D
P7	chr1	216061868	c.8123G>A	216061868	0.4972	nonsynonymous SNV	NM_206933	USH2A	p.G2708D
P7	chr17	29676270	c.7258+1G>C	29676270	0.1438	Splice_Site	NM_000267	NF1	-
P8	chr19	33793258	c.62_63insT	33793258	0.4853	frameshift insertion	NM_004364	CEBPA	p.S21fs
P8	chr11	32417916	c.1135_1136insACTCTTG	32417916	0.181	frameshift insertion	NM_024426	WT1	p.V379fs
P8	chr11	32456567	c.324_325insTCTCTCT	32456567	0.6961	frameshift insertion	NM_024426	WT1	p.A109fs
P8	chrX	100611207	c.1399C>G	100611207	0.4944	nonsynonymous SNV	NM_000061	BTK	p.Q467E
P8	chr19	33792414	c.907G>C	33792414	0.4538	nonsynonymous SNV	NM_004364	CEBPA	p.A303P
P8	chr1	36933434	c.1853C>T	36933434	0.2203	nonsynonymous SNV	NM_156039	CSF3R	p.T618I
P8	chr17	7750710	c.1197C>G	7750710	0.4338	nonsynonymous SNV	NM_001080424	KDM6B	p.S399R
P8	chr4	55599321	c.2447A>T	55599321	0.1483	nonsynonymous SNV	NM_000222	KIT	p.D816V
P8	chr4	106197285	c.5618T>C	106197285	0.5035	nonsynonymous SNV	NM_001127208	TET2	p.I1873T
P8	chr8	117878824	c.144+1G>T	117878824	0.0101	Splice_Site	NM_006265	RAD21	-
P9	chr19	33792391	c.929_930insCTC	33792391	0.9621	nonframeshift insertion	NM_004364	CEBPA	p.T310delinsTS
P9	chr14	73749106	c.1057C>T	73749106	0.5004	nonsynonymous SNV	NM_001005743	NUMB	p.P353S
P9	chr7	82532004	c.13491A>G	82532004	0.5245	nonsynonymous SNV	NM_033026	PCLO	p.I4497M
P9	chr7	82784441	c.1516T>C	82784441	0.0059	nonsynonymous SNV	NM_033026	PCLO	p.S506P
P9	chr7	82784438	c.1519A>G	82784438	0.0058	nonsynonymous SNV	NM_033026	PCLO	p.T507A
P10	chr17	48638951	c.131C>T	48638951	0.5174	nonsynonymous SNV	NM_018896	CACNA1G	p.A44V
P10	chr17	7749581	c.422C>T	7749581	0.4748	nonsynonymous SNV	NM_001080424	KDM6B	p.A141V
P10	chr13	48955457	c.1573G>A	48955457	0.4941	nonsynonymous SNV	NM_000321	RB1	p.A525T
P11	chr17	30325953	c.2151_2155del	30325957	0.4946	frameshift deletion	NM_015355	SUZ12	p.S717fs
P11	chr4	55599340	c.2466T>G	55599340	0.6026	nonsynonymous SNV	NM_000222	KIT	p.N822K
P11	chr5	67576373	c.652G>C	67576373	0.4886	nonsynonymous SNV	NM_181523	PIK3R1	p.E218Q
P11	chr19	1440233	c.305T>A	1440233	0.0394	nonsynonymous SNV	NM_001018	RPS15	p.F102Y
P12	chr6	26157138	c.520delA	26157138	0.0104	frameshift deletion	NM_005321	HIST1H1E	p.K174fs
P12	chr7	148512131	c.1531dupG	148512131	0.6043	frameshift insertion	NM_001203247	EZH2	p.D511fs
P12	chr11	32456261	c.630dupC	32456261	0.579	frameshift insertion	NM_024426	WT1	p.A211fs
P12	chr7	148526849	c.435_455del	148526869	0.203	nonframeshift deletion	NM_001203247	EZH2	p.145_152del
P12	chr12	11992215	c.305_306insTCTTCC	11992215	0.4197	nonframeshift insertion	NM_001987	ETV6	p.F102delinsFLP
P12	chr10	27311569	c.4130A>C	27311569	0.5789	nonsynonymous SNV	NM_001256053	ANKRD26	p.E1377A
P12	chr15	45007621	c.68G>A	45007621	0.4452	nonsynonymous SNV	NM_004048	B2M	p.R23H
P12	chr12	12037400	c.1031A>G	12037400	0.449	nonsynonymous SNV	NM_001987	ETV6	p.Y344C
P12	chr9	139399422	c.4721T>A	139399422	0.5657	nonsynonymous SNV	NM_017617	NOTCH1	p.L1574Q
P12	chr11	124747880	c.3034G>T	124747880	0.6333	nonsynonymous SNV	NM_022370	ROBO3	p.A1012S
P13	chr11	32417911	c.1140dupG	32417911	0.0071	frameshift insertion	NM_024426	WT1	p.S381fs

P13	chr13	28608252	c.1803_1804insGGCTCCGATTTCAGAGAATATGAATATGATCTC	28608252	0.0994	nonframeshift insertion	NM_004119	FLT3-ITD	p.K602delinsGSDFREYEYDLK
P13	chr17	48745060	c.1577G>A	48745060	0.5051	nonsynonymous SNV	NM_003786	ABCC3	p.R526H
P13	chr13	28592642	c.2503G>T	28592642	0.0083	nonsynonymous SNV	NM_004119	FLT3	p.D835Y
P13	chr22	37524563	c.1229C>G	37524563	0.4835	nonsynonymous SNV	NM_001346223	IL2RB	p.P410R
P13	chrX	53223671	c.3688T>G	53223671	0.4643	nonsynonymous SNV	NM_004187	KDM5C	p.S1230A
P13	chr11	118342626	c.752A>G	118342626	0.7513	nonsynonymous SNV	NM_001197104	KMT2A	p.D251G
P13	chr1	115258744	c.38G>A	115258744	0.0057	nonsynonymous SNV	NM_002524	NRAS	p.G13D
P13	chr11	32417914	c.1138C>G	32417914	0.0063	nonsynonymous SNV	NM_024426	WT1	p.R380G
P14	chr4	55589768	c.1248_1257delinsACTT	55589775	0.0818	frameshift deletion	NM_000222	KIT	p.T417_D419delinsL
P14	chr4	55589767	c.1249_1256delinsCACGC	55589774	0.0185	frameshift deletion	NM_000222	KIT	p.T417_D419delinsHA
P14	chr4	55589770	c.1252_1257delinsGGG	55589775	0.0142	frameshift deletion	NM_000222	KIT	p.Y418_D419delinsG
P14	chr2	30143117	c.409C>G	30143117	0.4837	nonsynonymous SNV	NM_004304	ALK	p.R137G
P14	chr13	28602340	c.2028C>A	28602340	0.2179	nonsynonymous SNV	NM_004119	FLT3	p.N676K
P14	chr4	55599334	c.2460T>A	55599334	0.0155	nonsynonymous SNV	NM_000222	KIT	p.D820E
P14	chr4	55599340	c.2466T>A	55599340	0.039	nonsynonymous SNV	NM_000222	KIT	p.N822K
P14	chr12	49436010	c.5971G>A	49436010	0.4937	nonsynonymous SNV	NM_003482	KMT2D	p.G1991S
P14	chr12	25398284	c.35G>T	25398284	0.0138	nonsynonymous SNV	NM_004985	KRAS	p.G12V
P14	chr16	30721409	c.1094C>T	30721409	0.4874	nonsynonymous SNV	NM_006662	SRCAP	p.P365L
P15	chr17	48701813	c.6322G>A	48701813	0.4801	nonsynonymous SNV	NM_018896	CACNA1G	p.A2108T
P15	chr22	41527475	c.1366G>A	41527475	0.4869	nonsynonymous SNV	NM_001429	EP300	p.V456I
P15	chr22	41523549	c.965G>C	41523549	0.4901	nonsynonymous SNV	NM_001429	EP300	p.G322A
P15	chr1	115256530	c.181C>A	115256530	0.2935	nonsynonymous SNV	NM_002524	NRAS	p.Q61K
P15	chr1	115256529	c.182A>T	115256529	0.0352	nonsynonymous SNV	NM_002524	NRAS	p.Q61L
P15	chr1	115258748	c.34G>T	115258748	0.0124	nonsynonymous SNV	NM_002524	NRAS	p.G12C
P15	chr1	115258747	c.35G>A	115258747	0.105	nonsynonymous SNV	NM_002524	NRAS	p.G12D
P15	chrX	39923850	c.3241G>T	39923850	0.4515	stopgain	NM_001123383	BCOR	p.E1081X
P16	chrX	39933593	c.1005dupC	39933593	0.3513	frameshift insertion	NM_001123383	BCOR	p.S336fs
P16	chr15	90631838	c.515G>A	90631838	0.4223	nonsynonymous SNV	NM_002168	IDH2	p.R172K
P16	chr2	25457290	c.2598-1G>A	25457290	0.4281	Splice_Site	NM_022552	DNMT3A	-
P17	chr8	3351160	c.1433G>C	3351160	0.4691	nonsynonymous SNV	NM_033225	CSMD1	p.R478T
P17	chr19	10897280	c.890G>A	10897280	0.4921	nonsynonymous SNV	NM_001005361	DNM2	p.R297H
P17	chr13	41240174	c.176C>T	41240174	0.4846	nonsynonymous SNV	NM_002015	FOXO1	p.P59L
P17	chr15	90631934	c.419G>A	90631934	0.0169	nonsynonymous SNV	NM_002168	IDH2	p.R140Q
P17	chr7	82544211	c.13091T>C	82544211	0.0088	nonsynonymous SNV	NM_033026	PCLO	p.L4364P
P17	chr1	216371722	c.4016T>C	216371722	0.5147	nonsynonymous SNV	NM_206933	USH2A	p.V1339A
P18	chr17	7750178	c.753_761del	7750186	0.5766	nonframeshift deletion	NM_001080424	KDM6B	p.251_254del
P18	chr17	48646326	c.338G>A	48646326	0.4982	nonsynonymous SNV	NM_018896	CACNA1G	p.R113H
P18	chr12	25398284	c.35G>T	25398284	0.0078	nonsynonymous SNV	NM_004985	KRAS	p.G12V
P18	chr1	115258747	c.35G>A	115258747	0.0063	nonsynonymous SNV	NM_002524	NRAS	p.G12D
P18	chr1	115258745	c.37G>C	115258745	0.3812	nonsynonymous SNV	NM_002524	NRAS	p.G13R
P18	chr16	30735363	c.4618A>C	30735363	0.482	nonsynonymous SNV	NM_006662	SRCAP	p.T1540P
P19	chr12	49430935	c.10202_10204del	49430937	0.4964	nonframeshift deletion	NM_003482	KMT2D	p.3401_3402del

P19	chr17	48678432	c.3812G>A	48678432	0.4968	nonsynonymous SNV	NM_018896	CACNA1G	p.R1271Q
P19	chr12	49436428	c.5783G>A	49436428	0.4851	nonsynonymous SNV	NM_003482	KMT2D	p.G1928D
P19	chr16	11348977	c.359C>T	11348977	0.495	nonsynonymous SNV	NM_003745	SOCS1	p.A120V
P20	chr19	33793074	c.247delC	33793074	0.4729	frameshift deletion	NM_004364	CEBPA	p.Q83fs
P20	chr19	33792382	c.937_939del	33792384	0.4747	nonframeshift deletion	NM_004364	CEBPA	p.313_313del
P20	chr7	148529784	c.305A>G	148529784	0.5246	nonsynonymous SNV	NM_001203247	EZH2	p.N102S
P20	chr7	50450300	c.484C>T	50450300	0.1139	nonsynonymous SNV	NM_006060	IKZF1	p.R162W
P20	chr4	55599321	c.2447A>T	55599321	0.2027	nonsynonymous SNV	NM_000222	KIT	p.D816V
P20	chr12	49421023	c.14726C>T	49421023	0.4994	nonsynonymous SNV	NM_003482	KMT2D	p.P4909L
P20	chr1	115256530	c.181C>A	115256530	0.0053	nonsynonymous SNV	NM_002524	NRAS	p.Q61K
P20	chr1	115258745	c.37G>C	115258745	0.1176	nonsynonymous SNV	NM_002524	NRAS	p.G13R
P20	chr13	49039470	c.2455C>G	49039470	0.4591	nonsynonymous SNV	NM_000321	RB1	p.L819V
P20	chrX	15841029	c.1113C>A	15841029	0.4912	nonsynonymous SNV	NM_005089	ZRSR2	p.D371E
P20	chr1	36932116	c.2434A>T	36932116	0.0145	stopgain	NM_156039	CSF3R	p.K812X
P21	chr19	33793122	c.198dupC	33793122	0.4808	frameshift insertion	NM_004364	CEBPA	p.Y67fs
P21	chr7	101891965	c.4161_4163del	101891967	0.4582	nonframeshift deletion	NM_181552	CUX1	p.1387_1388del
P21	chr19	17948746	c.1688_1696del	17948754	0.3527	nonframeshift deletion	NM_000215	JAK3	p.563_566del
P21	chr19	33792375	c.945_946insCTG	33792375	0.4631	nonframeshift insertion	NM_004364	CEBPA	p.E316delinsLE
P21	chr15	90631934	c.419G>A	90631934	0.028	nonsynonymous SNV	NM_002168	IDH2	p.R140Q
P21	chr1	39776025	c.3040G>C	39776025	0.4973	nonsynonymous SNV	NM_012090	MACF1	p.D1014H
P21	chr9	36966691	c.635A>C	36966691	0.4777	nonsynonymous SNV	NM_016734	PAX5	p.H212P
P21	chr19	1440233	c.305T>A	1440233	0.0247	nonsynonymous SNV	NM_001018	RPS15	p.F102Y
P21	chr11	64533436	c.1774T>G	64533436	0.4556	nonsynonymous SNV	NM_201995	SF1	p.S592A
P21	chr7	138266451	c.2626C>T	138266451	0.3995	nonsynonymous SNV	NM_003852	TRIM24	p.R876C
P22	chr19	33793252	c.68dupC	33793252	0.4423	frameshift insertion	NM_004364	CEBPA	p.P23fs
P22	chr19	33792397	c.923_924insCGT	33792397	0.4802	nonframeshift insertion	NM_004364	CEBPA	p.V308delinsVV
P22	chr4	24577882	c.491C>T	24577882	0.4322	nonsynonymous SNV	NM_001358	DHX15	p.S164F
P22	chr22	41553402	c.3491G>T	41553402	0.2577	nonsynonymous SNV	NM_001429	EP300	p.C1164F
P22	chr1	115256528	c.183A>T	115256528	0.2348	nonsynonymous SNV	NM_002524	NRAS	p.Q61H
P22	chr1	115258745	c.37G>C	115258745	0.0181	nonsynonymous SNV	NM_002524	NRAS	p.G13R
P23	chrX	44941872	c.3352A>G	44941872	0.4706	nonsynonymous SNV	NM_001291415	KDM6A	p.T1118A
P23	chr4	55599320	c.2446G>T	55599320	0.2153	nonsynonymous SNV	NM_000222	KIT	p.D816Y
P23	chr4	55599321	c.2447A>T	55599321	0.1131	nonsynonymous SNV	NM_000222	KIT	p.D816V
P23	chr1	115256530	c.181C>A	115256530	0.0214	nonsynonymous SNV	NM_002524	NRAS	p.Q61K
P23	chr1	115256528	c.183A>C	115256528	0.0462	nonsynonymous SNV	NM_002524	NRAS	p.Q61H
P23	chr16	8997215	c.1749G>A	8997215	0.4271	nonsynonymous SNV	NM_003470	USP7	p.M583I
P23	chr17	7705237	c.8874G>A	7705237	0.4474	stopgain	NM_020877	DNAH2	p.W2958X
P24	chr11	119148868	c.1096_1096del	119148876	0.0313	frameshift deletion	NM_005188	CBL	p.E366fs
P24	chr2	25966987	c.2218dupA	25966987	0.2697	frameshift insertion	NM_018263	ASXL2	p.T740fs
P24	chr8	117870630	c.441dupA	117870630	0.0111	frameshift insertion	NM_006265	RAD21	p.E148fs
P24	chr4	24572314	c.664C>G	24572314	0.1551	nonsynonymous SNV	NM_001358	DHX15	p.R222G
P24	chr3	47883111	c.673A>G	47883111	0.0079	nonsynonymous SNV	NM_138615	DHX30	p.S225G
P24	chr17	7751911	c.2305A>G	7751911	0.5496	nonsynonymous SNV	NM_001080424	KDM6B	p.K769E

P24	chr4	55599320	c.2446G>C	55599320	0.0186	nonsynonymous SNV	NM_000222	KIT	p.D816H
P24	chr4	55599322	c.2448C>A	55599322	0.0057	nonsynonymous SNV	NM_000222	KIT	p.D816E
P24	chr12	25398282	c.37G>C	25398282	0.0086	nonsynonymous SNV	NM_004985	KRAS	p.G13R
P24	chr1	115258744	c.38G>A	115258744	0.0071	nonsynonymous SNV	NM_002524	NRAS	p.G13D
P24	chr13	49039456	c.2441A>C	49039456	0.5029	nonsynonymous SNV	NM_000321	RB1	p.K814T
P24	chr9	5126790	c.3398G>C	5126790	0.4883	stoploss	NM_004972	JAK2	p.X1133S
P25	chr22	41566542	c.4419delG	41566542	0.4537	frameshift deletion	NM_001429	EP300	p.K1473fs
P25	chr13	28608262	c.1793_1794insTGATTTCAGAGAATATGA	28608262	0.4097	nonframeshift insertion	NM_004119	FLT3-ITD	p.E598delinsDDFREYE
P25	chr12	25398281	c.38G>A	25398281	0.017	nonsynonymous SNV	NM_004985	KRAS	p.G13D
P25	chr5	142779985	c.420G>T	142779985	0.472	nonsynonymous SNV	NM_000176	NR3C1	p.K140N
P25	chr11	124746281	c.2704G>A	124746281	0.5066	nonsynonymous SNV	NM_022370	ROBO3	p.G902R
P25	chr19	33793146	c.175G>T	33793146	0.491	stopgain	NM_004364	CEBPA	p.E59X
P26	chr17	29553477	c.2027dupC	29553477	0.5926	frameshift insertion	NM_000267	NF1	p.T676fs
P26	chr5	170837545	c.861_862insTGCA	170837545	0.4776	frameshift insertion	NM_002520	NPM1	p.L287fs
P26	chr10	27306636	c.4298T>C	27306636	0.4908	nonsynonymous SNV	NM_001256053	ANKRD26	p.L1433S
P26	chr6	157099260	c.197A>G	157099260	0.4776	nonsynonymous SNV	NM_017519	ARID1B	p.N66S
P26	chr14	99640795	c.2378G>A	99640795	0.0186	nonsynonymous SNV	NM_138576	BCL11B	p.R793H
P26	chr13	28592642	c.2503G>T	28592642	0.3037	nonsynonymous SNV	NM_004119	FLT3	p.D835Y
P26	chr21	44524453	c.104G>T	44524453	0.0212	nonsynonymous SNV	NM_006758	U2AF1	p.R35L
P26	chr1	215987206	c.9611A>G	215987206	0.4773	nonsynonymous SNV	NM_206933	USH2A	p.H3204R
P26	chr8	117870678	c.394C>T	117870678	0.2957	stopgain	NM_006265	RAD21	p.Q132X
P27	chr12	12006417	c.386dupT	12006417	0.4236	frameshift insertion	NM_001987	ETV6	p.L129fs
P27	chr12	12006433	c.401_402insTG	12006433	0.4263	frameshift insertion	NM_001987	ETV6	p.F134fs
P27	chr2	25457242	c.2645G>A	25457242	0.9516	nonsynonymous SNV	NM_022552	DNMT3A	p.R882H
P27	chr15	90631934	c.419G>A	90631934	0.4474	nonsynonymous SNV	NM_002168	IDH2	p.R140Q
P27	chr1	115258748	c.34G>T	115258748	0.0071	nonsynonymous SNV	NM_002524	NRAS	p.G12C
P27	chr1	115258747	c.35G>A	115258747	0.3704	nonsynonymous SNV	NM_002524	NRAS	p.G12D
P27	chrX	153628891	c.416G>A	153628891	0.0199	nonsynonymous SNV	NM_006013	RPL10	p.R139H
P27	chrX	53441953	c.275G>A	53441953	0.0099	nonsynonymous SNV	NM_006306	SMC1A	p.R92H
P28	chr2	25972917	c.1505_1508del	25972920	0.4567	frameshift deletion	NM_018263	ASXL2	p.E502fs
P28	chr2	25972912	c.1513delC	25972912	0.4555	frameshift deletion	NM_018263	ASXL2	p.H505fs
P28	chr16	9057112	c.30_31insCAG	9057112	0.4214	nonframeshift insertion	NM_003470	USP7	p.K11delinsQK
P28	chr2	29551299	c.1331G>T	29551299	0.4801	nonsynonymous SNV	NM_004304	ALK	p.W444L
P28	chr8	2836176	c.8524A>G	2836176	0.5019	nonsynonymous SNV	NM_033225	CSMD1	p.M2842V
P28	chr17	7756783	c.4993T>G	7756783	0.4272	nonsynonymous SNV	NM_001080424	KDM6B	p.F1665V
P28	chr4	55599320	c.2446G>C	55599320	0.4392	nonsynonymous SNV	NM_000222	KIT	p.D816H
P29	chr12	49433773	c.7780delC	49433773	0.0075	frameshift deletion	NM_003482	KMT2D	p.L2594fs
P29	chrX	123220424	c.3082dupT	123220424	0.2099	frameshift insertion	NM_001042749	STAG2	p.T1027fs
P29	chr16	30749516	c.8155_8157del	30749518	0.492	nonframeshift deletion	NM_006662	SRCAP	p.2719_2719del
P29	chr17	48697152	c.5890C>T	48697152	0.5279	nonsynonymous SNV	NM_018896	CACNA1G	p.P1964S
P29	chr13	103382694	c.20353T>C	103382694	0.5078	nonsynonymous SNV	NM_001146197	CCDC168	p.S6785P
P29	chr2	25470498	c.976C>A	25470498	0.1386	nonsynonymous SNV	NM_022552	DNMT3A	p.R326S
P30	chr2	25463184	c.2309C>T	25463184	0.0152	nonsynonymous SNV	NM_022552	DNMT3A	p.S770L

P30	chr22	41513598	c.502G>T	41513598	0.5035	nonsynonymous SNV	NM_001429	EP300	p.G168W
P30	chr1	216348605	c.4616C>T	216348605	0.5046	nonsynonymous SNV	NM_206933	USH2A	p.T1539I
P31	chr19	33793229	c.90dupC	33793229	0.4493	frameshift insertion	NM_004364	CEBPA	p.F31fs
P31	chr2	25457242	c.2645G>A	25457242	0.4295	nonsynonymous SNV	NM_022552	DNMT3A	p.R882H
P31	chr17	29657414	c.5647A>G	29657414	0.4317	nonsynonymous SNV	NM_000267	NF1	p.N1883D
P31	chr1	115258748	c.34G>T	115258748	0.0236	nonsynonymous SNV	NM_002524	NRAS	p.G12C
P31	chr1	115258747	c.35G>C	115258747	0.3345	nonsynonymous SNV	NM_002524	NRAS	p.G12A
P31	chr7	82784378	c.1579A>G	82784378	0.0071	nonsynonymous SNV	NM_033026	PCLO	p.T527A
P32	chr7	101892059	c.4255G>T	101892059	0.544	nonsynonymous SNV	NM_181552	CUX1	p.D1419Y
P32	chr2	209113112	c.395G>T	209113112	0.4466	nonsynonymous SNV	NM_001282386	IDH1	p.R132L
P32	chr22	37533659	c.505G>A	37533659	0.4553	nonsynonymous SNV	NM_001346223	IL2RB	p.A169T
P32	chr12	49443827	c.3544T>C	49443827	0.4963	nonsynonymous SNV	NM_003482	KMT2D	p.C1182R
P32	chrX	133547982	c.715C>T	133547982	0.9472	nonsynonymous SNV	NM_001015877	PHF6	p.H239Y
P32	chr6	138200348	c.1766G>A	138200348	0.0063	nonsynonymous SNV	NM_006290	TNFAIP3	p.G589D
P32	chr11	119149219	c.1228-1G>A	119149219	0.8073	Splice_Site	NM_005188	CBL	-
P33	chr20	31022441	c.1927dupG	31022441	0.3497	frameshift insertion	NM_015338	ASXL1	p.G642fs
P33	chr4	106157870	c.2771_2772insTGCG	106157870	0.0672	frameshift insertion	NM_001127208	TET2	p.H924fs
P33	chr13	103385999	c.17048A>G	103385999	0.0061	nonsynonymous SNV	NM_001146197	CCDC168	p.K5683R
P33	chr21	39763607	c.866A>G	39763607	0.338	nonsynonymous SNV	NM_001243432	ERG	p.K289R
P33	chr13	28592641	c.2504A>T	28592641	0.1119	nonsynonymous SNV	NM_004119	FLT3	p.D835V
P33	chr4	55592202	c.1526A>T	55592202	0.0327	nonsynonymous SNV	NM_000222	KIT	p.K509I
P33	chr4	55599333	c.2459A>G	55599333	0.0777	nonsynonymous SNV	NM_000222	KIT	p.D820G
P33	chr4	55599340	c.2466T>G	55599340	0.0225	nonsynonymous SNV	NM_000222	KIT	p.N822K
P33	chr12	25398284	c.35G>A	25398284	0.0323	nonsynonymous SNV	NM_004985	KRAS	p.G12D
P33	chr12	25378561	c.437C>T	25378561	0.0063	nonsynonymous SNV	NM_004985	KRAS	p.A146V
P33	chr1	115258744	c.38G>A	115258744	0.0522	nonsynonymous SNV	NM_002524	NRAS	p.G13D
P33	chr7	82595474	c.3630A>C	82595474	0.55	nonsynonymous SNV	NM_033026	PCLO	p.E1210D
P34	chr2	25965041	c.4165G>A	25965041	0.4919	nonsynonymous SNV	NM_018263	ASXL2	p.E1389K
P34	chr7	101840344	c.1653G>T	101840344	0.5124	nonsynonymous SNV	NM_181552	CUX1	p.M551I
P34	chr2	25466797	c.1906G>T	25466797	0.1811	nonsynonymous SNV	NM_022552	DNMT3A	p.V636L
P34	chr2	25457259	c.2628C>G	25457259	0.0284	nonsynonymous SNV	NM_022552	DNMT3A	p.D876E
P34	chr12	12022849	c.955A>G	12022849	0.4932	nonsynonymous SNV	NM_001987	ETV6	p.M319V
P34	chr2	209113113	c.394C>G	209113113	0.1805	nonsynonymous SNV	NM_001282386	IDH1	p.R132G
P34	chr7	151945124	c.2395A>G	151945124	0.0934	nonsynonymous SNV	NM_170606	KMT2C	p.N799D
P34	chr2	48028180	c.3058A>G	48028180	0.4956	nonsynonymous SNV	NM_000179	MSH6	p.N1020D
P34	chr9	139390941	c.7250C>G	139390941	0.4804	nonsynonymous SNV	NM_017617	NOTCH1	p.P2417R
P35	chr19	33793074	c.247delC	33793074	0.4672	frameshift deletion	NM_004364	CEBPA	p.Q83fs
P35	chr19	33792437	c.884C>G	33792437	0.4976	nonsynonymous SNV	NM_004364	CEBPA	p.A295G
P35	chr3	128200145	c.1160C>A	128200145	0.5049	nonsynonymous SNV	NM_032638	GATA2	p.T387N
P35	chr3	128202758	c.962T>A	128202758	0.501	nonsynonymous SNV	NM_032638	GATA2	p.L321H
P35	chr2	209113210	c.297A>G	209113210	0.4871	nonsynonymous SNV	NM_001282386	IDH1	p.I99M
P35	chr12	25398281	c.38G>A	25398281	0.0083	nonsynonymous SNV	NM_004985	KRAS	p.G13D
P35	chr5	142779689	c.716G>A	142779689	0.4958	nonsynonymous SNV	NM_000176	NR3C1	p.G239E

P35	chr1	115256528	c.183A>C	115256528	0.383	nonsynonymous SNV	NM_002524	NRAS	p.Q61H
P35	chr1	115258748	c.34G>T	115258748	0.046	nonsynonymous SNV	NM_002524	NRAS	p.G12C
P35	chr1	115258747	c.35G>A	115258747	0.0132	nonsynonymous SNV	NM_002524	NRAS	p.G12D
P35	chr11	124749141	c.3589A>G	124749141	0.4901	nonsynonymous SNV	NM_022370	ROBO3	p.I1197V
P35	chr7	101840058	c.1367T>G	101840058	0.1533	stopgain	NM_181552	CUX1	p.L456X
P36	chr19	33793074	c.247delC	33793074	0.4784	frameshift deletion	NM_004364	CEBPA	p.Q83fs
P36	chr6	41903745	c.568dupC	41903745	0.4778	frameshift insertion	NM_001136017	CCND3	p.R190fs
P36	chr19	33792404	c.916_917insAGC	33792404	0.4741	nonframeshift insertion	NM_004364	CEBPA	p.R306delinsQR
P36	chr7	148511174	c.1713C>G	148511174	0.9511	nonsynonymous SNV	NM_001203247	EZH2	p.C571W
P36	chr3	128202731	c.989G>T	128202731	0.4594	nonsynonymous SNV	NM_032638	GATA2	p.R330L
P36	chr6	18130918	c.719A>G	18130918	0.47	nonsynonymous SNV	NM_000367	TPMT	p.Y240C
P36	chrX	133527662	c.372C>A	133527662	0.9639	stopgain	NM_001015877	PHF6	p.Y124X
P37	chr6	157519997	c.4027C>T	157519997	0.0085	nonsynonymous SNV	NM_017519	ARID1B	p.P1343S
P37	chr19	1440233	c.305T>A	1440233	0.0411	nonsynonymous SNV	NM_001018	RPS15	p.F102Y
P37	chr4	106196223	c.4556G>T	106196223	0.511	nonsynonymous SNV	NM_001127208	TET2	p.G1519V
P38	chr22	41573885	c.6170_6171del	41573886	0.3598	frameshift deletion	NM_001429	EP300	p.T2057fs
P38	chr2	25972653	c.1771_1772insTGGGCTTTTT	25972653	0.0223	frameshift insertion	NM_018263	ASXL2	p.R591fs
P38	chr6	41903745	c.568dupC	41903745	0.4341	frameshift insertion	NM_001136017	CCND3	p.R190fs
P38	chr12	25378561	c.437C>T	25378561	0.3435	nonsynonymous SNV	NM_004985	KRAS	p.A146V
P39	chr4	187629117	c.1865G>A	187629117	0.4967	nonsynonymous SNV	NM_005245	FAT1	p.G622E
P39	chr4	55599340	c.2466T>G	55599340	0.3729	nonsynonymous SNV	NM_000222	KIT	p.N822K
P39	chr12	49443758	c.3613G>A	49443758	0.4919	nonsynonymous SNV	NM_003482	KMT2D	p.V1205I
P40	chr2	25973090	c.1331_1335del	25973094	0.4122	frameshift deletion	NM_018263	ASXL2	p.K444fs
P40	chr8	117866528	c.1116_1117del	117866529	0.0135	frameshift deletion	NM_006265	RAD21	p.L372fs
P40	chr11	47380498	c.386_390del	47380502	0.0076	frameshift deletion	NM_003120	SPI1	p.P129fs
P40	chr20	31024186	c.3671G>C	31024186	0.538	nonsynonymous SNV	NM_015338	ASXL1	p.R1224T
P40	chr4	55598066	c.2263G>A	55598066	0.4645	nonsynonymous SNV	NM_000222	KIT	p.A755T
P40	chr1	43818397	c.1862T>C	43818397	0.0074	nonsynonymous SNV	NM_005373	MPL	p.I621T
P40	chr17	29664865	c.6608A>G	29664865	0.4885	nonsynonymous SNV	NM_000267	NF1	p.K2203R
P41	chr2	25966454	c.2751_2752insGCTG	25966454	0.4572	frameshift insertion	NM_018263	ASXL2	p.S918fs
P41	chr13	28608256	c.1799_1800insATGTTTAGG TTTCAGAGAATATGAATAT GA	28608256	0.458	nonframeshift insertion	NM_004119	FLT3-ITD	p.D600delinsECLGF REYEYD
P41	chr17	48742514	c.1339A>G	48742514	0.5109	nonsynonymous SNV	NM_003786	ABCC3	p.N447D
P41	chr17	30264337	c.72_76delinsTCC	30264341	0.4632	nonsynonymous SNV	NM_015355	SUZ12	p.F25fs
P41	chr9	21971120	c.238C>T	21971120	0.9322	stopgain	NM_000077	CDKN2A	p.R80X
P42	chr8	4494912	c.254T>C	4494912	0.5208	nonsynonymous SNV	NM_033225	CSMD1	p.I85T
P42	chr2	209113113	c.394C>T	209113113	0.099	nonsynonymous SNV	NM_001282386	IDH1	p.R132C
P42	chr7	82545282	c.12020A>G	82545282	0.0065	nonsynonymous SNV	NM_033026	PCLO	p.N4007S
P42	chrX	39913165	c.4848T>A	39913165	0.0138	stopgain	NM_001123383	BCOR	p.Y1616X
P43	chr6	41903745	c.568dupC	41903745	0.1666	frameshift insertion	NM_001136017	CCND3	p.R190fs
P43	chr19	33793141	c.179dupC	33793141	0.444	frameshift insertion	NM_004364	CEBPA	p.T60fs
P43	chr6	26157054	c.437dupC	26157054	0.0954	frameshift insertion	NM_005321	HIST1H1E	p.T146fs
P43	chr19	33792375	c.946G>A	33792375	0.0188	nonsynonymous SNV	NM_004364	CEBPA	p.E316K

P43	chr4	187540811	c.6929C>G	187540811	0.4965	nonsynonymous SNV	NM_005245	FAT1	p.S2310C
P43	chr3	128202731	c.989G>T	128202731	0.407	nonsynonymous SNV	NM_032638	GATA2	p.R330L
P43	chr7	82785203	c.754C>T	82785203	0.2024	nonsynonymous SNV	NM_033026	PCLO	p.P252S
P44	chr19	33793229	c.91_92insCT	33793229	0.4217	frameshift insertion	NM_004364	CEBPA	p.F31fs
P44	chr5	170837545	c.859_860insTCTG	170837545	0.4229	frameshift insertion	NM_002520	NPM1	p.L287fs
P44	chr16	3778287	c.6761T>C	3778287	0.0058	nonsynonymous SNV	NM_004380	CREBBP	p.L2254P
P44	chr8	3443725	c.1155A>T	3443725	0.4844	nonsynonymous SNV	NM_033225	CSMD1	p.K385N
P44	chr8	3257030	c.2288C>T	3257030	0.4848	nonsynonymous SNV	NM_033225	CSMD1	p.T763I
P44	chr2	25457243	c.2644C>T	25457243	0.4677	nonsynonymous SNV	NM_022552	DNMT3A	p.R882C
P44	chr4	106196702	c.5035T>G	106196702	0.0141	nonsynonymous SNV	NM_001127208	TET2	p.Y1679D
P44	chr13	42793364	c.3214-2A>G	42793364	0.9328	Splice_Site	NM_001204504	DGKH	-
P44	chr13	28608268	c.1787_1788insGGGGCGTTGATTTCAGAGAATATGAATATGATCTCAAATGGGAGTTTCCAAGAGAAAATTTAGAGTTTGGTA	28608268	0.0597	stopgain	NM_004119	FLT3-ITD	p.E596delinsEGRX
P45	chr7	101892095	c.4291_4292insGCCCCGCGGCCCCGA	101892095	0.3986	nonframeshift insertion	NM_181552	CUX1	p.G1431delinsGPAAPS
P45	chr2	30143429	c.97G>T	30143429	0.4688	nonsynonymous SNV	NM_004304	ALK	p.A33S
P45	chr10	27349309	c.1529C>T	27349309	0.4723	nonsynonymous SNV	NM_001256053	ANKRD26	p.A510V
P45	chr17	7752005	c.2399C>T	7752005	0.4507	nonsynonymous SNV	NM_001080424	KDM6B	p.P800L
P45	chr11	118343445	c.1571A>G	118343445	0.5104	nonsynonymous SNV	NM_001197104	KMT2A	p.N524S
P45	chr1	115256529	c.182A>C	115256529	0.4437	nonsynonymous SNV	NM_002524	NRAS	p.Q61P
P45	chr16	11349292	c.44C>T	11349292	0.5036	nonsynonymous SNV	NM_003745	SOCS1	p.T15I
P45	chr6	18130918	c.719A>G	18130918	0.4882	nonsynonymous SNV	NM_000367	TPMT	p.Y240C
P46	chr7	50467998	c.1233delC	50467998	0.0075	frameshift deletion	NM_006060	IKZF1	p.L411fs
P46	chr1	43818345	c.1810_1813del	43818348	0.4814	frameshift deletion	NM_005373	MPL	p.L604fs
P46	chr19	33793252	c.68dupC	33793252	0.4031	frameshift insertion	NM_004364	CEBPA	p.P23fs
P46	chr7	50467818	c.1054dupA	50467818	0.1952	frameshift insertion	NM_006060	IKZF1	p.H351fs
P46	chr19	33792408	c.912_913insTTG	33792408	0.4208	nonframeshift insertion	NM_004364	CEBPA	p.Q305delinsLQ
P46	chr13	103397388	c.5659C>T	103397388	0.4874	stopgain	NM_001146197	CCDC168	p.Q1887X
P47	chr19	33793258	c.63_68delinsACCCC	33793258	0.4454	frameshift deletion	NM_004364	CEBPA	p.S21fs
P47	chr16	30747644	c.6853_6854insCTGGC	30747644	0.4328	frameshift insertion	NM_006662	SRCAP	p.A2285fs
P47	chr20	57429696	c.1376_1377insTGACGCCCCAGCCGATCCCGACTCCGGGGCGGCCCG	57429696	0.2272	nonframeshift insertion	NM_080425	GNAS	p.P459delinsPDAPADPDSGAAR
P47	chr7	82581760	c.8509A>G	82581760	0.489	nonsynonymous SNV	NM_033026	PCLO	p.R2837G
P47	chr4	106155534	c.435T>G	106155534	0.4824	nonsynonymous SNV	NM_001127208	TET2	p.S145R
P47	chr1	216166466	c.6701C>G	216166466	0.4968	nonsynonymous SNV	NM_206933	USH2A	p.A2234G
P47	chr19	33792467	c.853_854insG	33792467	0.4219	stopgain	NM_004364	CEBPA	p.Y285_R286delinsX
P48	chr2	25966455	c.2750_2751insAAATGACCGGGAAGACCCCCACT	25966455	0.4056	frameshift insertion	NM_018263	ASXL2	p.S917fs
P48	chr12	11992214	c.304_314delinsCTTCGCTATCGGCTGACT	11992214	0.4168	frameshift insertion	NM_001987	ETV6	p.F102fs
P48	chr5	35874600	c.756_757delinsGGAGGTGC	35874601	0.39165	frameshift insertion	NM_002185	IL7R	p.V253delinsEVL
P48	chrX	133527587	c.297_298insAC	133527587	0.8849	frameshift insertion	NM_001015877	PHF6	p.C99fs
P48	chr12	111884760	c.849_850insAC	111884760	0.7762	frameshift insertion	NM_005475	SH2B3	p.T283fs
P48	chr10	27326862	c.2494C>T	27326862	0.4984	nonsynonymous SNV	NM_001256053	ANKRD26	p.L832F
P48	chr3	47859578	c.95A>G	47859578	0.449	nonsynonymous SNV	NM_138615	DHX30	p.N32S
P48	chr13	41133899	c.1729G>A	41133899	0.4371	nonsynonymous SNV	NM_002015	FOXO1	p.G577S

P48	chr9	139397762	c.5039T>C	139397762	0.4186	nonsynonymous SNV	NM_017617	NOTCH1	p.I1680T
P49	chr15	91328250	c.2762G>C	91328250	0.4752	nonsynonymous SNV	NM_000057	BLM	p.G921A
P49	chr6	41903754	c.560A>C	41903754	0.0088	nonsynonymous SNV	NM_001136017	CCND3	p.K187T
P49	chr8	4494883	c.283C>G	4494883	0.4703	nonsynonymous SNV	NM_033225	CSMD1	p.Q95E
P49	chr15	90631838	c.515G>A	90631838	0.2366	nonsynonymous SNV	NM_002168	IDH2	p.R172K
P49	chr15	88680721	c.536A>G	88680721	0.0072	nonsynonymous SNV	NM_001012338	NTRK3	p.E179G
P49	chrX	133527597	c.308dupA	133527597	0.2721	stopgain	NM_001015877	PHF6	p.Y103_H104delinsX
P50	chr2	25973137	c.1288_1289insGTCG	25973137	0.4504	frameshift insertion	NM_018263	ASXL2	p.E430fs
P50	chr11	69466028	c.866_867insCGTGCGCCC	69466028	0.008	nonframeshift insertion	NM_053056	CCND1	p.D289delinsDVRP
P50	chr17	48677128	c.3598A>G	48677128	0.5033	nonsynonymous SNV	NM_018896	CACNA1G	p.N1200D
P50	chr7	50450366	c.550C>T	50450366	0.0204	nonsynonymous SNV	NM_006060	IKZF1	p.R184W
P50	chr12	25398281	c.38G>A	25398281	0.1709	nonsynonymous SNV	NM_004985	KRAS	p.G13D
P50	chr1	115256530	c.181C>A	115256530	0.18	nonsynonymous SNV	NM_002524	NRAS	p.Q61K
P50	chr18	42530413	c.1108G>A	42530413	0.4968	nonsynonymous SNV	NM_015559	SETBP1	p.E370K
P50	chrX	123210203	c.2555C>T	123210203	0.4262	nonsynonymous SNV	NM_001042749	STAG2	p.A852V
P50	chr14	103369758	c.1127G>A	103369758	0.5127	nonsynonymous SNV	NM_003300	TRAF3	p.R376Q
P51	chr17	29527460	c.909_910insCT	29527460	0.0124	frameshift insertion	NM_000267	NF1	p.L303fs
P51	chr2	25457243	c.2644C>T	25457243	0.4905	nonsynonymous SNV	NM_022552	DNMT3A	p.R882C
P51	chr13	28592642	c.2503G>T	28592642	0.1665	nonsynonymous SNV	NM_004119	FLT3	p.D835Y
P51	chr4	55599321	c.2447A>T	55599321	0.2556	nonsynonymous SNV	NM_000222	KIT	p.D816V
P52	chr12	49444946	c.2520_2522delinsGCG	49444944	0.0051	frameshift deletion	NM_003482	KMT2D	p.P840_C841delinsPH
P52	chr13	28608308	c.1747_1748insGCTCCTCAGATAATGAGTACTTCTACGTTGATTTCAGAGAATATGAATATGATCTCAAATGGGAGTTTCCAAGAGAAAATTTAGAG	28608308	0.4157	frameshift insertion	NM_004119	FLT3-ITD	p.G583fs
P52	chr5	170837545	c.859_860insTCTG	170837545	0.4021	frameshift insertion	NM_002520	NPM1	p.L287fs
P52	chrX	76938062	c.2686G>A	76938062	0.005	nonsynonymous SNV	NM_000489	ATRX	p.D896N
P52	chr8	3263672	c.2143C>T	3263672	0.4761	nonsynonymous SNV	NM_033225	CSMD1	p.L715F
P52	chr15	90631934	c.419G>A	90631934	0.4527	nonsynonymous SNV	NM_002168	IDH2	p.R140Q
P52	chr15	88680721	c.536A>G	88680721	0.0078	nonsynonymous SNV	NM_001012338	NTRK3	p.E179G
P52	chr4	187538991	c.8749C>T	187538991	0.3776	stopgain	NM_005245	FAT1	p.R2917X
P53	chr2	25466784	c.1919T>C	25466784	0.2245	nonsynonymous SNV	NM_022552	DNMT3A	p.F640S
P53	chr2	25463293	c.2200T>C	25463293	0.2683	nonsynonymous SNV	NM_022552	DNMT3A	p.F734L
P53	chr4	187542860	c.4880G>A	187542860	0.5164	nonsynonymous SNV	NM_005245	FAT1	p.R1627Q
P53	chr20	57429816	c.1496C>G	57429816	0.5106	nonsynonymous SNV	NM_080425	GNAS	p.A499G
P53	chr2	209113113	c.394C>T	209113113	0.2827	nonsynonymous SNV	NM_001282386	IDH1	p.R132C
P53	chr5	149502735	c.2053C>T	149502735	0.5027	nonsynonymous SNV	NM_002609	PDGFRB	p.R685C
P53	chr16	11348977	c.359C>T	11348977	0.4697	nonsynonymous SNV	NM_003745	SOCS1	p.A120V
P53	chr4	106157539	c.2440C>T	106157539	0.4919	nonsynonymous SNV	NM_001127208	TET2	p.R814C
P53	chrX	39933455	c.1144G>T	39933455	0.2208	stopgain	NM_001123383	BCOR	p.E382X
P53	chrX	129162789	c.4258C>T	129162789	0.2322	stopgain	NM_021946	BCORL1	p.R1420X
P54	chr5	170837545	c.859_860insTCTG	170837545	0.4831	frameshift insertion	NM_002520	NPM1	p.L287fs
P54	chr6	157099332	c.269_270insCCA	157099332	0.4964	nonframeshift insertion	NM_017519	ARID1B	p.A90delinsAH
P54	chr2	25457243	c.2644C>T	25457243	0.4685	nonsynonymous SNV	NM_022552	DNMT3A	p.R882C

P54	chr15	90631934	c.419G>A	90631934	0.4357	nonsynonymous SNV	NM_002168	IDH2	p.R140Q
P54	chr12	25398285	c.34G>A	25398285	0.4181	nonsynonymous SNV	NM_004985	KRAS	p.G12S
P54	chr13	28608218	c.1837+1G>A	28608218	0.0229	Splice_Site	NM_004119	FLT3	-
P54	chr13	28608218	c.1837+1->ATAATGAGTACTTCTACGTTGATTTCAGAGAATATGAATATGATCTCAAATGGGAGTTTCCAAGAGAAAATTTAGAGTTTG	28608218	0.4634	Splice_Site	NM_004119	FLT3-ITD	-
P55	chr19	33793252	c.68dupC	33793252	0.4694	frameshift insertion	NM_004364	CEBPA	p.P23fs
P55	chr19	33792392	c.928_929insAGA	33792392	0.4958	nonframeshift insertion	NM_004364	CEBPA	p.T310delinsKT
P55	chr7	101892095	c.4291_4292insGCCCCGCGGCCCCGA	101892095	0.4139	nonframeshift insertion	NM_181552	CUX1	p.G1431delinsGPAAPS
P55	chr4	187541130	c.6610G>A	187541130	0.4936	nonsynonymous SNV	NM_005245	FAT1	p.V2204M
P55	chr3	128202761	c.959G>A	128202761	0.0421	nonsynonymous SNV	NM_032638	GATA2	p.G320D
P55	chr3	128202759	c.961C>G	128202759	0.4408	nonsynonymous SNV	NM_032638	GATA2	p.L321V
P55	chr12	25378647	c.351A>T	25378647	0.0315	nonsynonymous SNV	NM_004985	KRAS	p.K117N
P55	chr1	115258744	c.38G>A	115258744	0.2361	nonsynonymous SNV	NM_002524	NRAS	p.G13D
P55	chr9	36923394	c.868G>A	36923394	0.0213	nonsynonymous SNV	NM_016734	PAX5	p.G290R
P55	chr12	112888202	c.218C>T	112888202	0.0071	nonsynonymous SNV	NM_002834	PTPN11	p.T73I
P55	chr16	30722981	c.1408G>A	30722981	0.4551	nonsynonymous SNV	NM_006662	SRCAP	p.V470I
P56	chr2	25462010	c.2397delC	25462010	0.0146	frameshift deletion	NM_022552	DNMT3A	p.P799fs
P56	chr2	25966987	c.2219_2220insCCCC	25966987	0.0471	frameshift insertion	NM_018263	ASXL2	p.T740fs
P56	chr2	25966985	c.2220dupG	25966985	0.2428	frameshift insertion	NM_018263	ASXL2	p.R741fs
P56	chr4	55599320	c.2446G>C	55599320	0.0071	nonsynonymous SNV	NM_000222	KIT	p.D816H
P56	chr4	55599340	c.2466T>G	55599340	0.1808	nonsynonymous SNV	NM_000222	KIT	p.N822K
P56	chr15	88680721	c.536A>G	88680721	0.0072	nonsynonymous SNV	NM_001012338	NTRK3	p.E179G
P57	chr5	170837545	c.859_860insTCTG	170837545	0.4403	frameshift insertion	NM_002520	NPM1	p.L287fs
P57	chr13	28608223	c.1832_1833insATATGATCTCAAATGGGAGTTTCCAAGAGAAAATTTAGA	28608223	0.2177	nonframeshift insertion	NM_004119	FLT3-ITD	p.E611delinsEYDLKWEFPRENLE
P57	chr2	25457242	c.2645G>A	25457242	0.4324	nonsynonymous SNV	NM_022552	DNMT3A	p.R882H
P57	chr4	106190848	c.4126G>A	106190848	0.4075	nonsynonymous SNV	NM_001127208	TET2	p.D1376N
P58	chr19	10897321	c.931G>A	10897321	0.4922	nonsynonymous SNV	NM_001005361	DNM2	p.V311M
P58	chr15	90631838	c.515G>A	90631838	0.4361	nonsynonymous SNV	NM_002168	IDH2	p.R172K
P58	chr17	7749988	c.641T>G	7749988	0.4851	nonsynonymous SNV	NM_001080424	KDM6B	p.L214R
P58	chrX	133547992	c.725G>T	133547992	0.9122	nonsynonymous SNV	NM_001015877	PHF6	p.C242F
P58	chr16	81946232	c.1965G>C	81946232	0.4845	nonsynonymous SNV	NM_002661	PLCG2	p.E655D
P58	chr16	30733971	c.3794C>A	30733971	0.5164	nonsynonymous SNV	NM_006662	SRCAP	p.P1265Q
P59	chr2	25967121	c.2085delA	25967121	0.2314	frameshift deletion	NM_018263	ASXL2	p.P695fs
P59	chr2	25457242	c.2645G>A	25457242	0.2426	nonsynonymous SNV	NM_022552	DNMT3A	p.R882H
P59	chr2	209113113	c.394C>T	209113113	0.2271	nonsynonymous SNV	NM_001282386	IDH1	p.R132C
P60	chr5	170837545	c.861_862insTGTA	170837545	0.4624	frameshift insertion	NM_002520	NPM1	p.L287fs
P60	chr13	28608262	c.1793_1794insCGAGGTGACCGGCTCCTCAGATAATGAGTACTTCTACGTTGATTTCAGAGAATATGA	28608262	0.4739	nonframeshift insertion	NM_004119	FLT3-ITD	p.E598delinsDEVTGSSDNEYFYVDFREYE
P60	chr2	25457243	c.2644C>T	25457243	0.4501	nonsynonymous SNV	NM_022552	DNMT3A	p.R882C
P60	chr13	28608262	c.1794A>C	28608262	0.0128	nonsynonymous SNV	NM_004119	FLT3	p.E598D
P60	chr12	49421006	c.14743T>C	49421006	0.3886	nonsynonymous SNV	NM_003482	KMT2D	p.S4915P
P60	chr17	29527515	c.964A>G	29527515	0.4912	nonsynonymous SNV	NM_000267	NF1	p.I322V

P60	chr11	32438072	c.965G>A	32438072	0.4832	nonsynonymous SNV	NM_024426	WT1	p.S322N
P61	chr1	215814037	c.14831delT	215814037	0.4945	frameshift deletion	NM_206933	USH2A	p.L4944fs
P61	chr20	31024311	c.3796C>G	31024311	0.4907	nonsynonymous SNV	NM_015338	ASXL1	p.L1266V
P61	chr1	115256528	c.183A>T	115256528	0.068	nonsynonymous SNV	NM_002524	NRAS	p.Q61H
P61	chr1	115258744	c.38G>A	115258744	0.0083	nonsynonymous SNV	NM_002524	NRAS	p.G13D
P61	chr3	47142966	c.4997A>C	47142966	0.3919	nonsynonymous SNV	NM_014159	SETD2	p.Y1666S
P61	chr12	111885484	c.1261C>T	111885484	0.4992	nonsynonymous SNV	NM_005475	SH2B3	p.R421W
P62	chr2	25464480	c.2033delA	25464480	0.4487	frameshift deletion	NM_022552	DNMT3A	p.Q678fs
P62	chr7	50367242	c.49_50insGC	50367242	0.4476	frameshift insertion	NM_006060	IKZF1	p.S17fs
P62	chr17	7751859	c.2253_2264del	7751870	0.6296	nonframeshift deletion	NM_001080424	KDM6B	p.751_755del
P62	chr1	36933434	c.1853C>T	36933434	0.45	nonsynonymous SNV	NM_156039	CSF3R	p.T618I
P62	chr7	101559479	c.115C>T	101559479	0.5082	nonsynonymous SNV	NM_181552	CUX1	p.R39W
P62	chr2	25470498	c.976C>T	25470498	0.4809	nonsynonymous SNV	NM_022552	DNMT3A	p.R326C
P62	chr9	139399389	c.4754T>C	139399389	0.4199	nonsynonymous SNV	NM_017617	NOTCH1	p.L1585P
P62	chr11	64536914	c.647A>C	64536914	0.4912	nonsynonymous SNV	NM_201995	SF1	p.K216T
P62	chr16	11348789	c.547G>A	11348789	0.4717	nonsynonymous SNV	NM_003745	SOCS1	p.V183M
P62	chr1	36932213	c.2337delT	36932213	0.9476	stopgain	NM_156039	CSF3R	p.Y779X
P63	chr6	157099890	c.827delC	157099890	0.0097	frameshift deletion	NM_017519	ARID1B	p.A276fs
P63	chrX	39933296	c.1303delG	39933296	0.5331	frameshift deletion	NM_001123383	BCOR	p.D435fs
P63	chr12	12038941	c.1234delG	12038941	0.0108	frameshift deletion	NM_001987	ETV6	p.G412fs
P63	chr7	82784833	c.1123_1124insCTCTTGGTCCTGCTAAGCCTCCAGCTCAGC	82784833	0.4874	nonframeshift insertion	NM_033026	PCLO	p.Q375delinsPLGPAKPPAQQ
P63	chr17	7660447	c.1943A>T	7660447	0.5014	nonsynonymous SNV	NM_020877	DNAH2	p.E648V
P63	chr12	25398285	c.34G>A	25398285	0.2111	nonsynonymous SNV	NM_004985	KRAS	p.G12S
P63	chr12	25398284	c.35G>A	25398284	0.0185	nonsynonymous SNV	NM_004985	KRAS	p.G12D
P63	chr21	44524456	c.101C>T	44524456	0.2389	nonsynonymous SNV	NM_006758	U2AF1	p.S34F
P64	chr5	170837545	c.859_860insTCTG	170837545	0.3781	frameshift insertion	NM_002520	NPM1	p.L287fs
P64	chr13	28608244	c.1811_1812insCTACGTTGATTTCAGAGAATATGAATATGATCTCAAATGGGA	28608244	0.5791	nonframeshift insertion	NM_004119	FLT3-ITD	p.E604delinsDYVDFREYEYDLKWE
P64	chr2	25457242	c.2645G>A	25457242	0.3915	nonsynonymous SNV	NM_022552	DNMT3A	p.R882H
P64	chr13	28608244	c.1812G>C	28608244	0.0218	nonsynonymous SNV	NM_004119	FLT3	p.E604D
P64	chr7	138269563	c.2918A>T	138269563	0.4932	nonsynonymous SNV	NM_003852	TRIM24	p.E973V
P65	chr7	82784700	c.1257delA	82784700	0.0154	frameshift deletion	NM_033026	PCLO	p.K419fs
P65	chr11	32413541	c.1408_1409insAGACCCACA	32413541	0.2044	nonframeshift insertion	NM_024426	WT1	p.T470delinsKTHT
P65	chr8	3256944	c.2374C>G	3256944	0.5549	nonsynonymous SNV	NM_033225	CSMD1	p.P792A
P65	chr15	90631838	c.515G>A	90631838	0.1859	nonsynonymous SNV	NM_002168	IDH2	p.R172K
P65	chr12	57501504	c.139G>A	57501504	0.5027	nonsynonymous SNV	NM_003153	STAT6	p.A47T
P65	chrX	53423537	c.2563G>T	53423537	0.0399	stopgain	NM_006306	SMC1A	p.E855X
P66	chr19	33793154	c.166dupT	33793154	0.4536	frameshift insertion	NM_004364	CEBPA	p.C56fs
P66	chr22	41574722	c.7007_7009del	41574724	0.471	nonframeshift deletion	NM_001429	EP300	p.2336_2337del
P66	chr6	157099981	c.918_919insGGC	157099981	0.4402	nonframeshift insertion	NM_017519	ARID1B	p.A306delinsAG
P66	chr19	33792431	c.890G>C	33792431	0.429	nonsynonymous SNV	NM_004364	CEBPA	p.R297P
P66	chr16	3778102	c.6946C>T	3778102	0.4967	nonsynonymous SNV	NM_004380	CREBBP	p.P2316S
P66	chr7	148526910	c.394C>T	148526910	0.8806	nonsynonymous SNV	NM_001203247	EZH2	p.P132S

P66	chr2	209113112	c.395G>A	209113112	0.4567	nonsynonymous SNV	NM_001282386	IDH1	p.R132H
P66	chr4	106196231	c.4564A>G	106196231	0.4919	nonsynonymous SNV	NM_001127208	TET2	p.M1522V
P67	chrX	44929459	c.2715_2718del	44929462	0.0347	frameshift deletion	NM_001291415	KDM6A	p.T905fs
P67	chr13	28608284	c.1771_1772insACGTTGATTTCAGAGAATATGAATATGATCTCAAATGGGAGTTTCCAAGAGAAAATTTAGAGTTTGGTAAGAATGGAATGTGCCAA	28608284	0.4389	frameshift insertion	NM_004119	FLT3-ITD	p.Y591fs
P67	chr20	57429696	c.1376_1377insTGACGCCCCAGCCGATCCCGACTCCGGGGCGGCCCG	57429696	0.4707	nonframeshift insertion	NM_080425	GNAS	p.P459delinsPDAPADPDSGAAR
P67	chr22	22221708	c.22_23insCGGCGG	22221708	0.5032	nonframeshift insertion	NM_002745	MAPK1	p.G8delinsAAG
P67	chr10	64573994	c.404G>T	64573994	0.4962	nonsynonymous SNV	NM_000399	EGR2	p.S135I
P67	chr7	148512600	c.1529A>G	148512600	0.0064	nonsynonymous SNV	NM_001203247	EZH2	p.K510R
P67	chr4	55599320	c.2446G>C	55599320	0.0291	nonsynonymous SNV	NM_000222	KIT	p.D816H
P67	chr4	55599321	c.2447A>T	55599321	0.0102	nonsynonymous SNV	NM_000222	KIT	p.D816V
P67	chr4	55599340	c.2466T>A	55599340	0.0223	nonsynonymous SNV	NM_000222	KIT	p.N822K
P68	chr6	157099425	c.362_363insGC	157099425	0.3456	frameshift insertion	NM_017519	ARID1B	p.Q121fs
P68	chr6	157100097	c.1034C>T	157100097	0.5017	nonsynonymous SNV	NM_017519	ARID1B	p.A345V
P68	chr2	25965148	c.4058A>G	25965148	0.4865	nonsynonymous SNV	NM_018263	ASXL2	p.N1353S
P68	chr4	55599342	c.2468A>G	55599342	0.0881	nonsynonymous SNV	NM_000222	KIT	p.Y823C
P68	chr7	151945057	c.2462C>T	151945057	0.101	nonsynonymous SNV	NM_170606	KMT2C	p.P821L
P68	chr12	25398284	c.35G>A	25398284	0.328	nonsynonymous SNV	NM_004985	KRAS	p.G12D
P68	chr4	106158380	c.3281A>G	106158380	0.479	nonsynonymous SNV	NM_001127208	TET2	p.K1094R
P68	chr11	32413610	c.1340G>T	32413610	0.2986	nonsynonymous SNV	NM_024426	WT1	p.G447V
P68	chr2	25966986	c.2219_2220insTGCCGGGTAGCCC	25966986	0.3424	stopgain	NM_018263	ASXL2	p.T740_R741delinsTAGX
P69	chr11	32417914	c.1138_1140delinsGGGGG	32417914	0.2695	frameshift insertion	NM_024426	WT1	p.R380fs
P69	chr1	115256528	c.183A>C	115256528	0.2777	nonsynonymous SNV	NM_002524	NRAS	p.Q61H
P69	chr1	115258747	c.35G>A	115258747	0.3151	nonsynonymous SNV	NM_002524	NRAS	p.G12D
P70	chr11	32456580	c.311_312del	32456581	0.051	frameshift deletion	NM_024426	WT1	p.P104fs
P70	chr13	28608255	c.1800_1801insTTCAGAGAATATGAATATGAT	28608255	0.2134	nonframeshift insertion	NM_004119	FLT3-ITD	p.L601delinsFREYEYDL
P70	chr3	47883111	c.673A>G	47883111	0.0057	nonsynonymous SNV	NM_138615	DHX30	p.S225G
P70	chr6	18130918	c.719A>G	18130918	0.5131	nonsynonymous SNV	NM_000367	TPMT	p.Y240C
P71	chr6	157527697	c.5383C>T	157527697	0.4589	nonsynonymous SNV	NM_017519	ARID1B	p.R1795C
P71	chr13	42773989	c.2437A>C	42773989	0.0292	nonsynonymous SNV	NM_001204504	DGKH	p.T813P
P71	chr15	90631934	c.419G>A	90631934	0.4371	nonsynonymous SNV	NM_002168	IDH2	p.R140Q
P71	chr12	112926249	c.1382C>G	112926249	0.4104	nonsynonymous SNV	NM_002834	PTPN11	p.A461G
P72	chr3	47162529	c.3596_3597del	47162530	0.012	frameshift deletion	NM_014159	SETD2	p.Q1199fs
P72	chr11	102195395	c.155G>A	102195395	0.4772	nonsynonymous SNV	NM_001165	BIRC3	p.R52H
P72	chr15	88680721	c.536A>G	88680721	0.0079	nonsynonymous SNV	NM_001012338	NTRK3	p.E179G
P72	chr12	112888198	c.214G>A	112888198	0.4307	nonsynonymous SNV	NM_002834	PTPN11	p.A72T
P72	chr3	47163843	c.2283G>A	47163843	0.4948	nonsynonymous SNV	NM_014159	SETD2	p.M761I
P72	chr4	106157539	c.2440C>T	106157539	0.4933	nonsynonymous SNV	NM_001127208	TET2	p.R814C
P72	chr3	47162531	c.3595C>T	47162531	0.0121	stopgain	NM_014159	SETD2	p.Q1199X
P73	chr2	25467164	c.1711delG	25467164	0.0096	frameshift deletion	NM_022552	DNMT3A	p.A571fs
P73	chr13	103401551	c.1495dupA	103401551	0.4796	frameshift insertion	NM_001146197	CCDC168	p.T499fs

P73	chr3	128205089	c.351_352insCCCACCACCACAACCCCTGGACC	128205089	0.0996	frameshift insertion	NM_032638	GATA2	p.V118fs
P73	chr13	28592635	c.2508_2510del	28592637	0.0379	nonframeshift deletion	NM_004119	FLT3	p.836_837del
P73	chr11	119148966	c.1186T>C	119148966	0.0121	nonsynonymous SNV	NM_005188	CBL	p.C396R
P73	chr13	28608338	c.1718A>G	28608338	0.0637	nonsynonymous SNV	NM_004119	FLT3	p.E573G
P73	chr13	28608098	c.1868A>T	28608098	0.0802	nonsynonymous SNV	NM_004119	FLT3	p.K623I
P73	chr13	28602340	c.2028C>A	28602340	0.0263	nonsynonymous SNV	NM_004119	FLT3	p.N676K
P73	chr13	28592640	c.2505T>A	28592640	0.0101	nonsynonymous SNV	NM_004119	FLT3	p.D835E
P73	chrX	53223703	c.3656T>G	53223703	0.0626	nonsynonymous SNV	NM_004187	KDM5C	p.L1219R
P73	chr4	55599320	c.2446G>T	55599320	0.0132	nonsynonymous SNV	NM_000222	KIT	p.D816Y
P73	chr1	115258744	c.38G>T	115258744	0.0162	nonsynonymous SNV	NM_002524	NRAS	p.G13V
P73	chr18	42532738	c.3433G>A	42532738	0.4945	nonsynonymous SNV	NM_015559	SETBP1	p.G1145S
P74	chr15	91303933	c.1330T>A	91303933	0.4612	nonsynonymous SNV	NM_000057	BLM	p.C444S
P74	chr22	41574815	c.7100C>T	41574815	0.3501	nonsynonymous SNV	NM_001429	EP300	p.P2367L
P74	chr1	39853910	c.9210C>G	39853910	0.4901	nonsynonymous SNV	NM_012090	MACF1	p.F3070L
P74	chr15	88680721	c.536A>G	88680721	0.006	nonsynonymous SNV	NM_001012338	NTRK3	p.E179G
P74	chr1	216371893	c.3845G>T	216371893	0.4362	nonsynonymous SNV	NM_206933	USH2A	p.R1282I
P75	chr6	157099403	c.340_363del	157099426	0.4611	nonframeshift deletion	NM_017519	ARID1B	p.114_121del
P75	chr15	88680721	c.536A>G	88680721	0.0063	nonsynonymous SNV	NM_001012338	NTRK3	p.E179G
P75	chr16	30750833	c.9472C>T	30750833	0.4485	stopgain	NM_006662	SRCAP	p.Q3158X
P76	chr17	7727987	c.11795C>T	7727987	0.4549	nonsynonymous SNV	NM_020877	DNAH2	p.P3932L
P76	chr14	73753958	c.515G>A	73753958	0.5342	nonsynonymous SNV	NM_001005743	NUMB	p.R172Q
P77	chr6	26157138	c.520delA	26157138	0.0077	frameshift deletion	NM_005321	HIST1H1E	p.K174fs
P77	chr5	170837545	c.859_860insTCTG	170837545	0.4898	frameshift insertion	NM_002520	NPM1	p.L287fs
P77	chrX	39931690	c.2909C>T	39931690	0.0058	nonsynonymous SNV	NM_001123383	BCOR	p.A970V
P77	chr7	101891841	c.4037G>A	101891841	0.4947	nonsynonymous SNV	NM_181552	CUX1	p.G1346D
P77	chr2	25464456	c.2057A>G	25464456	0.475	nonsynonymous SNV	NM_022552	DNMT3A	p.D686G
P77	chr15	90631934	c.419G>A	90631934	0.4734	nonsynonymous SNV	NM_002168	IDH2	p.R140Q
P77	chr13	28608217	c.1837+2->AGAATATGAATATGATCTCAAATGGGAGTTTCCAAGAGAAAATTTAGAGTTTGG	28608217	0.4657	Splice_Site	NM_004119	FLT3-ITD	-
P77	chr1	215844544	c.13903C>T	215844544	0.0052	stopgain	NM_206933	USH2A	p.Q4635X
P78	chr8	117862975	c.1501_1502delinsAGGG(p.P501fs)	117862976	0.01435	frameshift deletion	NM_006265	RAD21	p.P501fs
P78	chr11	32417941	c.1110dupT	32417941	0.062	frameshift insertion	NM_024426	WT1	p.V371fs
P78	chr11	32417922	c.1129_1130insATTTCTCCTT	32417922	0.4794	frameshift insertion	NM_024426	WT1	p.T377fs
P78	chr13	28592630	c.2514_2515insCCC	28592630	0.0074	nonframeshift insertion	NM_004119	FLT3	p.D839delinsPD
P78	chr2	29940525	c.706C>G	29940525	0.5034	nonsynonymous SNV	NM_004304	ALK	p.P236A
P78	chr13	28592642	c.2503G>T	28592642	0.3224	nonsynonymous SNV	NM_004119	FLT3	p.D835Y
P78	chr3	128200720	c.1085G>A	128200720	0.3702	nonsynonymous SNV	NM_032638	GATA2	p.R362Q
P78	chr4	106197269	c.5602C>T	106197269	0.0794	nonsynonymous SNV	NM_001127208	TET2	p.H1868Y
P78	chr11	32417922	c.1131_1157delinsC	32417896	0.0091	stopgain	NM_024426	WT1	p.L378X
P79	chrX	44942752	c.3488delG	44942752	0.8068	frameshift deletion	NM_001291415	KDM6A	p.R1163fs
P79	chr8	117859772	c.1862dupT	117859772	0.021	frameshift insertion	NM_006265	RAD21	p.I621fs
P79	chr4	55599320	c.2446G>T	55599320	0.381	nonsynonymous SNV	NM_000222	KIT	p.D816Y
P79	chr8	128750986	c.523G>A	128750986	0.4973	nonsynonymous SNV	NM_002467	MYC	p.G175S

P79	chr7	82595515	c.3589G>A	82595515	0.0054	nonsynonymous SNV	NM_033026	PCLO	p.E1197K
P79	chr3	47163001	c.3125C>A	47163001	0.4733	nonsynonymous SNV	NM_014159	SETD2	p.S1042Y
P79	chr3	128205776	c.99C>A	128205776	0.014	stopgain	NM_032638	GATA2	p.Y33X
P80	chr11	32417911	c.1142_1143insCGGTC	32417911	0.4745	frameshift insertion	NM_024426	WT1	p.S381fs
P80	chr13	28608252	c.1803_1804insTACGTTGATTTCAGAGAATATGAATATGATCTC	28608252	0.2747	nonframeshift insertion	NM_004119	FLT3-ITD	p.K602delinsYVDFREYEYDLK
P81	chr19	33793207	c.103delC	33793216	0.2881	frameshift deletion	NM_004364	CEBPA	p.R35fs
P81	chr7	50468270	c.1505_1511del	50468276	0.0264	frameshift deletion	NM_006060	IKZF1	p.R502fs
P81	chr19	33792398	c.921_923del	33792400	0.2275	nonframeshift deletion	NM_004364	CEBPA	p.307_308del
P81	chr19	33793214	c.107G>C	33793214	0.2881	nonsynonymous SNV	NM_004364	CEBPA	p.G36A
P81	chr12	49437499	c.5386C>T	49437499	0.4805	nonsynonymous SNV	NM_003482	KMT2D	p.R1796W
P81	chr7	50455090	c.637C>T	50455090	0.0189	stopgain	NM_006060	IKZF1	p.R213X
P82	chr7	50455116	c.663delG	50455116	0.4425	frameshift deletion	NM_006060	IKZF1	p.E221fs
P82	chr12	49444946	c.2520_2522delinsGCG	49444944	0.0067	frameshift deletion	NM_003482	KMT2D	p.P840_C841delinsPH
P82	chr16	30732729	c.3473_3483del	30732739	0.4693	frameshift deletion	NM_006662	SRCAP	p.A1158fs
P82	chr19	33793119	c.201_202insCTAC	33793119	0.4859	frameshift insertion	NM_004364	CEBPA	p.I68fs
P82	chr19	33792382	c.934_939del	33792387	0.4448	nonframeshift deletion	NM_004364	CEBPA	p.312_313del
P82	chr13	28608262	c.1793_1794insTAATGAGTACTTCTACGTTGATTTCAGAGAATATGA	28608262	0.4536	nonframeshift insertion	NM_004119	FLT3-ITD	p.E598delinsDNEYFYVDFREYE
P82	chr13	73346009	c.1529G>A	73346009	0.4701	nonsynonymous SNV	NM_014953	DIS3	p.S510N
P82	chr7	148512600	c.1529A>G	148512600	0.0143	nonsynonymous SNV	NM_001203247	EZH2	p.K510R
P82	chr11	124738914	c.377C>A	124738914	0.4942	nonsynonymous SNV	NM_022370	ROBO3	p.P126Q
P82	chr4	106157539	c.2440C>T	106157539	0.5224	nonsynonymous SNV	NM_001127208	TET2	p.R814C
P83	chr4	55589768	c.1250_1256delinsT	55589774	0.0171	frameshift deletion	NM_000222	KIT	p.T417_D419delinsI
P83	chr17	29527531	c.980_981del	29527532	0.006	frameshift deletion	NM_000267	NF1	p.L327fs
P83	chr11	119148886	c.1106A>G	119148886	0.0248	nonsynonymous SNV	NM_005188	CBL	p.E369G
P83	chr16	3778287	c.6761T>C	3778287	0.0062	nonsynonymous SNV	NM_004380	CREBBP	p.L2254P
P83	chr4	55599320	c.2446G>T	55599320	0.1051	nonsynonymous SNV	NM_000222	KIT	p.D816Y
P83	chr1	115256529	c.182A>C	115256529	0.0205	nonsynonymous SNV	NM_002524	NRAS	p.Q61P
P83	chr1	115258747	c.35G>A	115258747	0.0051	nonsynonymous SNV	NM_002524	NRAS	p.G12D
P84	chr19	33793074	c.247delC	33793074	0.4072	frameshift deletion	NM_004364	CEBPA	p.Q83fs
P84	chr6	41903722	c.591dupC	41903722	0.0068	frameshift insertion	NM_001136017	CCND3	p.S198fs
P84	chr19	33792387	c.917_934del	33792404	0.3731	nonframeshift deletion	NM_004364	CEBPA	p.306_312del
P84	chr15	93467570	c.82G>A	93467570	0.5069	nonsynonymous SNV	NM_001271	CHD2	p.A28T
P84	chr3	128202770	c.950A>T	128202770	0.4096	nonsynonymous SNV	NM_032638	GATA2	p.N317I
P84	chr1	115256530	c.181C>A	115256530	0.1448	nonsynonymous SNV	NM_002524	NRAS	p.Q61K
P84	chr7	82584450	c.5819T>A	82584450	0.0176	nonsynonymous SNV	NM_033026	PCLO	p.V1940E
P85	chr11	32417944	c.1109_1110insGGCCCCAC	32417944	0.0127	frameshift insertion	NM_024426	WT1	p.R370fs
P85	chr11	32417914	c.1138delinsAA	32417914	0.023	frameshift insertion	NM_024426	WT1	p.R380fs
P85	chr11	32417911	c.1140dupG	32417911	0.016	frameshift insertion	NM_024426	WT1	p.S381fs
P85	chr11	32417911	c.1142_1143insAGCTTGTACGGTC	32417911	0.0063	frameshift insertion	NM_024426	WT1	p.S381fs
P85	chr11	32414249	c.1301_1302insATG	32414249	0.006	nonframeshift insertion	NM_024426	WT1	p.R434delinsRC
P85	chrX	15841230	c.1314_1315insAGCCGG	15841230	0.4925	nonframeshift insertion	NM_005089	ZRSR2	p.G438delinsGSR

P85	chr14	99641023	c.2150C>T	99641023	0.0074	nonsynonymous SNV	NM_138576	BCL11B	p.A717V
P85	chr1	36933434	c.1853C>T	36933434	0.2366	nonsynonymous SNV	NM_156039	CSF3R	p.T618I
P85	chr17	7690346	c.6598G>A	7690346	0.4866	nonsynonymous SNV	NM_020877	DNAH2	p.E2200K
P85	chr4	55599321	c.2447A>T	55599321	0.1066	nonsynonymous SNV	NM_000222	KIT	p.D816V
P85	chr7	151859375	c.11287G>A	151859375	0.4867	nonsynonymous SNV	NM_170606	KMT2C	p.A3763T
P85	chr11	32417910	c.1142C>A	32417910	0.0065	stopgain	NM_024426	WT1	p.S381X
P86	chr13	28608219	c.1836_1837insTGGTGGGACGTTGATTTCAGAGAATATGAATATGATCTCAAATGGGAGTTTCCAAGAGAAAATTTAGAGTTT	28608219	0.3897	nonframeshift insertion	NM_004119	FLT3-ITD	p.G613delinsWWDVDFREYEYDLKWEFPRENLEFG
P86	chr13	28608219	c.1837G>T	28608219	0.022	nonsynonymous SNV	NM_004119	FLT3	p.G613W
P86	chr1	115256528	c.183A>T	115256528	0.0803	nonsynonymous SNV	NM_002524	NRAS	p.Q61H
P86	chr7	82784486	c.1464_1471delinsACCCCCAT	82784486	0.00545	nonsynonymous SNV	NM_033026	PCLO	p.P491S
P87	chr19	33792294	c.1027delC	33792294	0.4517	frameshift deletion	NM_004364	CEBPA	p.R343fs
P87	chr11	32417925	c.1127_1136delinsGGG	32417916	0.0063	frameshift deletion	NM_024426	WT1	p.P376fs
P87	chr19	33792640	c.680_681insACGCC	33792640	0.025	frameshift insertion	NM_004364	CEBPA	p.P227fs
P87	chr11	32417941	c.1110dupT	32417941	0.0143	frameshift insertion	NM_024426	WT1	p.V371fs
P87	chr11	32417914	c.1138_1139insAC	32417914	0.046	frameshift insertion	NM_024426	WT1	p.R380fs
P87	chr11	32417914	c.1139_1140insAAACCACTCTTGTACG	32417914	0.0063	frameshift insertion	NM_024426	WT1	p.R380fs
P87	chr11	32417911	c.1140dupG	32417911	0.0401	frameshift insertion	NM_024426	WT1	p.S381fs
P87	chr8	38287284	c.274G>A	38287284	0.4883	nonsynonymous SNV	NM_015850	FGFR1	p.V92M
P87	chr12	25398284	c.35G>A	25398284	0.3179	nonsynonymous SNV	NM_004985	KRAS	p.G12D
P87	chr8	128750684	c.221C>T	128750684	0.3887	nonsynonymous SNV	NM_002467	MYC	p.P74L
P87	chr11	47377080	c.511C>T	47377080	0.0078	nonsynonymous SNV	NM_003120	SPI1	p.R171C
P87	chr11	32417910	c.1142C>A	32417910	0.011	stopgain	NM_024426	WT1	p.S381X
P88	chr5	170837545	c.859_860insTCTG	170837545	0.4711	frameshift insertion	NM_002520	NPM1	p.L287fs
P88	chr13	28608251	c.1804_1805insGAGAATATGAATATGATCTCA	28608251	0.5288	nonframeshift insertion	NM_004119	FLT3-ITD	p.K602delinsREYEYDLK
P88	chr2	25457242	c.2645G>A	25457242	0.4796	nonsynonymous SNV	NM_022552	DNMT3A	p.R882H
P88	chr4	55593464	c.1621A>T	55593464	0.4862	nonsynonymous SNV	NM_000222	KIT	p.M541L
P88	chr1	115258744	c.38G>A	115258744	0.081	nonsynonymous SNV	NM_002524	NRAS	p.G13D
P88	chr19	33792778	c.543C>A	33792778	0.2202	stopgain	NM_004364	CEBPA	p.Y181X
P88	chr16	30745044	c.6419delG	30745044	0.4686	stopgain	NM_006662	SRCAP	p.W2140X
P89	chr4	55589768	c.1248_1257delinsCTCCTA	55589775	0.3668	frameshift deletion	NM_000222	KIT	p.L416_D419delinsLL
P89	chr12	49445207	c.2258_2259insACCCCGGCCTGAGGAGCCGCACCTGTC	49445207	0.4813	nonframeshift insertion	NM_003482	KMT2D	p.S753delinsSPRPEEPHLS
P89	chr16	67671605	c.2014C>G	67671605	0.4888	nonsynonymous SNV	NM_006565	CTCF	p.Q672E
P89	chr4	55599321	c.2447A>T	55599321	0.031	nonsynonymous SNV	NM_000222	KIT	p.D816V
P89	chr20	35545163	c.1024G>A	35545163	0.4694	nonsynonymous SNV	NM_015474	SAMHD1	p.E342K
P90	chr19	33793210	c.99_111del	33793222	0.4813	frameshift deletion	NM_004364	CEBPA	p.F33fs
P90	chr19	33792371	c.949_950insAGAAGGTGCTGGAGC	33792371	0.4688	nonframeshift insertion	NM_004364	CEBPA	p.L317delinsQKVLEL
P90	chr3	128202800	c.919_920insACCGGC	128202800	0.0437	nonframeshift insertion	NM_032638	GATA2	p.R307delinsHRR
P90	chr3	128202759	c.961C>T	128202759	0.4091	nonsynonymous SNV	NM_032638	GATA2	p.L321F
P90	chr15	90631934	c.419G>A	90631934	0.0153	nonsynonymous SNV	NM_002168	IDH2	p.R140Q
P90	chr1	39818779	c.5114A>G	39818779	0.4976	nonsynonymous SNV	NM_012090	MACF1	p.N1705S
P90	chr11	124738998	c.461G>C	124738998	0.5133	nonsynonymous SNV	NM_022370	ROBO3	p.S154T

P90	chr19	1440233	c.305T>A	1440233	0.0374	nonsynonymous SNV	NM_001018	RPS15	p.F102Y
P90	chr22	41536259	c.1876C>T	41536259	0.1006	stopgain	NM_001429	EP300	p.R626X
P91	chr4	55589830	c.1312A>G	55589830	0.5051	nonsynonymous SNV	NM_000222	KIT	p.I438V
P91	chr4	55599320	c.2446G>C	55599320	0.1125	nonsynonymous SNV	NM_000222	KIT	p.D816H
P91	chr4	55599321	c.2447A>T	55599321	0.3481	nonsynonymous SNV	NM_000222	KIT	p.D816V
P91	chr12	49435009	c.6544G>T	49435009	0.5079	nonsynonymous SNV	NM_003482	KMT2D	p.A2182S
P91	chr12	49434871	c.6682A>G	49434871	0.4998	nonsynonymous SNV	NM_003482	KMT2D	p.T2228A
P92	chr19	33793252	c.68dupC	33793252	0.4431	frameshift insertion	NM_004364	CEBPA	p.P23fs
P92	chr19	33792755	c.558_566del	33792763	0.5375	nonframeshift deletion	NM_004364	CEBPA	p.186_189del
P92	chr8	3008979	c.5971C>T	3008979	0.4952	nonsynonymous SNV	NM_033225	CSMD1	p.P1991S
P92	chr3	128202767	c.953C>T	128202767	0.0085	nonsynonymous SNV	NM_032638	GATA2	p.A318V
P93	chr19	33792978	c.340_343del	33792981	0.4455	frameshift deletion	NM_004364	CEBPA	p.G114fs
P93	chr11	32417945	c.1106_1107del	32417946	0.0944	frameshift deletion	NM_024426	WT1	p.R369fs
P93	chr6	157099981	c.918_919insGGC	157099981	0.4464	nonframeshift insertion	NM_017519	ARID1B	p.A306delinsAG
P93	chr19	33792384	c.936_937insCAG	33792384	0.4551	nonframeshift insertion	NM_004364	CEBPA	p.K313delinsQK
P93	chrX	76939135	c.1613T>G	76939135	0.5069	nonsynonymous SNV	NM_000489	ATRX	p.V538G
P93	chr17	48653097	c.1334G>A	48653097	0.4726	nonsynonymous SNV	NM_018896	CACNA1G	p.R445H
P93	chr7	148512600	c.1529A>G	148512600	0.0059	nonsynonymous SNV	NM_001203247	EZH2	p.K510R
P93	chr4	187630465	c.517G>A	187630465	0.0057	nonsynonymous SNV	NM_005245	FAT1	p.A173T
P93	chr3	128202758	c.962T>C	128202758	0.4366	nonsynonymous SNV	NM_032638	GATA2	p.L321P
P93	chr4	55593661	c.1727T>C	55593661	0.0112	nonsynonymous SNV	NM_000222	KIT	p.L576P
P93	chr1	115256528	c.183A>T	115256528	0.0397	nonsynonymous SNV	NM_002524	NRAS	p.Q61H
P93	chr4	106164916	c.3784C>T	106164916	0.4308	nonsynonymous SNV	NM_001127208	TET2	p.R1262W
P94	chr2	25966987	c.2219_2220insTCCTT	25966987	0.3792	frameshift insertion	NM_018263	ASXL2	p.T740fs
P94	chrX	44929013	c.2270dupC	44929013	0.0082	frameshift insertion	NM_001291415	KDM6A	p.T757fs
P94	chrX	44945218	c.3698G>A	44945218	0.3946	nonsynonymous SNV	NM_001291415	KDM6A	p.C1233Y
P94	chr4	55599340	c.2466T>A	55599340	0.3867	nonsynonymous SNV	NM_000222	KIT	p.N822K
P94	chrX	70466326	c.2413A>C	70466326	0.0602	nonsynonymous SNV	NM_001171162	ZMYM3	p.T805P
P95	chr5	170837545	c.859_860insTCTG	170837545	0.4825	frameshift insertion	NM_002520	NPM1	p.L287fs
P95	chr3	47058712	c.7565_7566insCCCTGAA	47058712	0.1088	frameshift insertion	NM_014159	SETD2	p.K2522fs
P95	chr13	28608255	c.1800_1801insGATAATGAG TACTTCTACGTTGATTTCA GAGAATATGAATATGAT	28608255	0.4502	nonframeshift insertion	NM_004119	FLT3-ITD	p.L601delinsDNEY FYVDFREYEYDL
P95	chr2	30143169	c.357G>T	30143169	0.5319	nonsynonymous SNV	NM_004304	ALK	p.E119D
P95	chr15	90631934	c.419G>A	90631934	0.4646	nonsynonymous SNV	NM_002168	IDH2	p.R140Q
P95	chr11	118348892	c.3545G>A	118348892	0.0051	nonsynonymous SNV	NM_001197104	KMT2A	p.R1182H
P95	chr12	49427364	c.11124C>G	49427364	0.5064	nonsynonymous SNV	NM_003482	KMT2D	p.S3708R
P95	chr8	128750678	c.215C>A	128750678	0.0064	nonsynonymous SNV	NM_002467	MYC	p.P72H
P95	chr3	47164804	c.1322G>A	47164804	0.5067	nonsynonymous SNV	NM_014159	SETD2	p.R441Q
P96	chr12	49444944	c.2521_2522del	49444945	0.0066	frameshift deletion	NM_003482	KMT2D	p.C841fs
P96	chr19	33793114	c.206_207insTCGA	33793114	0.4457	frameshift insertion	NM_004364	CEBPA	p.D69fs
P96	chr12	49444946	c.2519_2520insGC	49444946	0.0067	frameshift insertion	NM_003482	KMT2D	p.P840fs
P96	chr19	10930663	c.1679_1681del	10930665	0.0066	nonframeshift deletion	NM_001005361	DNM2	p.560_561del
P96	chr19	33792413	c.907_908insTTG	33792413	0.4505	nonframeshift insertion	NM_004364	CEBPA	p.A303delinsVA

P96	chr19	10922986	c.1604_1605insAGGCGGCTC	10922986	0.1362	nonframeshift insertion	NM_001005361	DNM2	p.K535delinsKGGS
P96	chr19	10922991	c.1609G>A	10922991	0.0065	nonsynonymous SNV	NM_001005361	DNM2	p.G537S
P96	chr19	17945969	c.1970G>A	17945969	0.0061	nonsynonymous SNV	NM_000215	JAK3	p.R657Q
P96	chr1	115256530	c.181C>A	115256530	0.0109	nonsynonymous SNV	NM_002524	NRAS	p.Q61K
P96	chr1	115258747	c.35G>A	115258747	0.0545	nonsynonymous SNV	NM_002524	NRAS	p.G12D
P96	chrX	15339680	c.1403A>G	15339680	0.4883	nonsynonymous SNV	NM_002641	PIGA	p.Y468C
P96	chr19	1440030	c.102G>A	1440030	0.0337	nonsynonymous SNV	NM_001018	RPS15	p.M34I
P96	chr4	106193778	c.4240C>A	106193778	0.3013	nonsynonymous SNV	NM_001127208	TET2	p.Q1414K
P97	chr3	128205794	c.69_85delinsGGC	128205806	0.02455	frameshift deletion	NM_032638	GATA2	p.D23fs
P97	chrX	44928845	c.2101_2102insTAAGGGGA	44928845	0.0244	frameshift insertion	NM_001291415	KDM6A	p.S701fs
P97	chr4	106155554	c.455_456insA	106155554	0.0095	frameshift insertion	NM_001127208	TET2	p.S152fs
P97	chr11	32417914	c.1138_1141delinsGGGGT	32417914	0.45565	frameshift insertion	NM_024426	WT1	p.R380fs
P97	chr8	3046470	c.5462C>T	3046470	0.4854	nonsynonymous SNV	NM_033225	CSMD1	p.P1821L
P97	chr19	10939818	c.2165T>C	10939818	0.5038	nonsynonymous SNV	NM_001005361	DNM2	p.M722T
P97	chr15	66727455	c.171G>T	66727455	0.0718	nonsynonymous SNV	NM_002755	MAP2K1	p.K57N
P97	chr15	88680721	c.536A>G	88680721	0.0059	nonsynonymous SNV	NM_001012338	NTRK3	p.E179G
P97	chr4	106190822	c.4100C>A	106190822	0.0136	nonsynonymous SNV	NM_001127208	TET2	p.P1367Q
P97	chr11	32449502	c.872G>T	32449502	0.4521	nonsynonymous SNV	NM_024426	WT1	p.S291I
P97	chr13	28608217	c.1837+2->GCTGAGAGAATATGAATATGATCTCAAATGGGAGTTTCCAAGAGAAAATTTAGAGTTTGG	28608217	0.2829	Splice_Site	NM_004119	FLT3-ITD	-
P97	chr4	106193775	c.4237G>T	106193775	0.0159	stopgain	NM_001127208	TET2	p.E1413X
P97	chr4	106193967	c.4429G>T	106193967	0.0241	stopgain	NM_001127208	TET2	p.E1477X
P97	chr4	106194045	c.4507G>T	106194045	0.0065	stopgain	NM_001127208	TET2	p.E1503X
P98	chr19	33793100	c.221delA	33793100	0.4606	frameshift deletion	NM_004364	CEBPA	p.N74fs
P98	chr19	33792390	c.930_931insAAGCAG	33792390	0.4463	nonframeshift insertion	NM_004364	CEBPA	p.Q311delinsKQQ
P98	chr2	5833884	c.1031C>T	5833884	0.4382	nonsynonymous SNV	NM_003108	SOX11	p.S344F
P98	chr4	106197309	c.5642A>G	106197309	0.0073	nonsynonymous SNV	NM_001127208	TET2	p.H1881R
P99	chr19	33792390	c.930_931insACG	33792390	0.9305	nonframeshift insertion	NM_004364	CEBPA	p.Q311delinsTQ
P99	chrX	39934426	c.173C>T	39934426	0.0082	nonsynonymous SNV	NM_001123383	BCOR	p.A58V
P99	chr13	41240013	c.337C>T	41240013	0.531	nonsynonymous SNV	NM_002015	FOXO1	p.P113S
P99	chr2	48030700	c.3314G>A	48030700	0.4764	nonsynonymous SNV	NM_000179	MSH6	p.G1105E
P99	chr7	82584450	c.5819T>A	82584450	0.0105	nonsynonymous SNV	NM_033026	PCLO	p.V1940E
P100	chr4	55589771	c.1253_1254insCTTCCT	55589771	0.2148	nonframeshift insertion	NM_000222	KIT	p.Y418delinsYFL
P100	chr7	101740703	c.328G>A	101740703	0.4747	nonsynonymous SNV	NM_181552	CUX1	p.D110N
P100	chr4	55599320	c.2446G>T	55599320	0.0223	nonsynonymous SNV	NM_000222	KIT	p.D816Y
P100	chr4	55599340	c.2466T>G	55599340	0.0163	nonsynonymous SNV	NM_000222	KIT	p.N822K
P100	chr7	151945124	c.2395A>G	151945124	0.0847	nonsynonymous SNV	NM_170606	KMT2C	p.N799D
P100	chr1	115256529	c.182A>G	115256529	0.1124	nonsynonymous SNV	NM_002524	NRAS	p.Q61R
P100	chr1	115256528	c.183A>C	115256528	0.0174	nonsynonymous SNV	NM_002524	NRAS	p.Q61H
P100	chr1	115258744	c.38G>A	115258744	0.0129	nonsynonymous SNV	NM_002524	NRAS	p.G13D
P100	chr3	47163843	c.2283G>A	47163843	0.4978	nonsynonymous SNV	NM_014159	SETD2	p.M761I
P100	chrX	76939375	c.1373C>G	76939375	0.472	stopgain	NM_000489	ATRX	p.S458X
P101	chr12	49420826	c.14923C>T	49420826	0.47	nonsynonymous SNV	NM_003482	KMT2D	p.R4975C

P102	chr5	170837545	c.859_860insTCTG	170837545	0.4745	frameshift insertion	NM_002520	NPM1	p.L287fs
P102	chr13	28608223	c.1832_1833insATATGAATA TGATCTCAAATGGGAGTTT CCAAGAGAAAATTTAGA	28608223	0.4753	nonframeshift insertion	NM_004119	FLT3-ITD	p.E611delinsEYEY DLKWEFPRENLE
P102	chr2	29416743	c.4210C>G	29416743	0.5023	nonsynonymous SNV	NM_004304	ALK	p.L1404V
P102	chr2	25457243	c.2644C>T	25457243	0.4578	nonsynonymous SNV	NM_022552	DNMT3A	p.R882C
P102	chr2	48027746	c.2624T>C	48027746	0.4778	nonsynonymous SNV	NM_000179	MSH6	p.M875T
P102	chr1	115256529	c.182A>G	115256529	0.0062	nonsynonymous SNV	NM_002524	NRAS	p.Q61R
P102	chr16	30721032	c.832C>G	30721032	0.4928	nonsynonymous SNV	NM_006662	SRCAP	p.P278A
P103	chr9	5073770	c.1849G>T	5073770	0.8908	nonsynonymous SNV	NM_004972	JAK2	p.V617F
P103	chr20	35545189	c.998G>A	35545189	0.9252	nonsynonymous SNV	NM_015474	SAMHD1	p.R333H
P103	chr2	198267359	c.1998G>T	198267359	0.4518	nonsynonymous SNV	NM_012433	SF3B1	p.K666N
P104	chr11	32417914	c.1138_1139insCAAC	32417914	0.1169	frameshift insertion	NM_024426	WT1	p.R380fs
P104	chr11	32417914	c.1138_1140delinsGGGG	32417914	0.0819	frameshift insertion	NM_024426	WT1	p.R380fs
P104	chr19	13054634	c.1161T>G	13054634	0.503	nonsynonymous SNV	NM_004343	CALR	p.D387E
P104	chr12	49418361	c.16052G>A	49418361	0.0378	nonsynonymous SNV	NM_003482	KMT2D	p.R5351Q
P104	chr21	44514776	c.471G>T	44514776	0.0202	nonsynonymous SNV	NM_006758	U2AF1	p.Q157H
P105	chr19	33793080	c.241delC	33793080	0.8914	frameshift deletion	NM_004364	CEBPA	p.L81fs
P105	chr2	25462010	c.2397delC	25462010	0.0086	frameshift deletion	NM_022552	DNMT3A	p.P799fs
P105	chrX	15821895	c.288_289insAA	15821895	0.8924	frameshift insertion	NM_005089	ZRSR2	p.A96fs
P105	chr4	187629385	c.1597G>A	187629385	0.5069	nonsynonymous SNV	NM_005245	FAT1	p.V533I
P105	chr1	120458297	c.7048A>G	120458297	0.4969	nonsynonymous SNV	NM_024408	NOTCH2	p.S2350G
P105	chr19	1440233	c.305T>A	1440233	0.0446	nonsynonymous SNV	NM_001018	RPS15	p.F102Y
P106	chr13	28608262	c.1793_1794insCTACGTTGA TTTCAGAGAATATGA	28608262	0.1003	nonframeshift insertion	NM_004119	FLT3-ITD	p.E598delinsDYVD FREYE
P106	chr1	115258748	c.34G>A	115258748	0.0113	nonsynonymous SNV	NM_002524	NRAS	p.G12S
P106	chr1	115258747	c.35G>A	115258747	0.2716	nonsynonymous SNV	NM_002524	NRAS	p.G12D
P106	chr6	18130918	c.719A>G	18130918	0.5148	nonsynonymous SNV	NM_000367	TPMT	p.Y240C
P106	chr2	25463288	c.2205C>A	25463288	0.9528	stopgain	NM_022552	DNMT3A	p.Y735X
P107	chr5	170837545	c.859_860insTCTG	170837545	0.4606	frameshift insertion	NM_002520	NPM1	p.L287fs
P107	chr13	28608338	c.1717_1718insAAAGCCAGC TACAGATGGTACAGGTGA CCGGCTCCTCAGATAATGA GTACTTCTACGTTGATTTC AGAGAATATGAATATGAT CTC	28608338	0.5269	nonframeshift insertion	NM_004119	FLT3-ITD	p.E573delinsESQLQ MVQVTGSSDNEY FYVDFREYEYDL KWEFPRENLEFG KNE
P107	chrX	15841230	c.1314_1315insAGCCGG	15841230	0.5196	nonframeshift insertion	NM_005089	ZRSR2	p.G438delinsGSR
P107	chr20	31023472	c.2957A>G	31023472	0.4906	nonsynonymous SNV	NM_015338	ASXL1	p.N986S
P107	chr2	25463562	c.2120G>A	25463562	0.4434	nonsynonymous SNV	NM_022552	DNMT3A	p.G707D
P107	chr13	28608342	c.1714T>A	28608342	0.0304	nonsynonymous SNV	NM_004119	FLT3	p.Y572N
P107	chr8	117875483	c.160C>T	117875483	0.3843	nonsynonymous SNV	NM_006265	RAD21	p.R54W
P107	chr11	32417910	c.1142C>A	32417910	0.226	stopgain	NM_024426	WT1	p.S381X
P108	chr6	18148135	c.149_152del	18148138	0.467	frameshift deletion	NM_000367	TPMT	p.K50fs
P108	chr20	57428605	c.285C>G	57428605	0.4912	nonsynonymous SNV	NM_080425	GNAS	p.S95R
P108	chr12	49420991	c.14758C>T	49420991	0.607	nonsynonymous SNV	NM_003482	KMT2D	p.P4920S
P108	chr1	115258747	c.35G>A	115258747	0.4674	nonsynonymous SNV	NM_002524	NRAS	p.G12D
P108	chrX	133549137	c.821G>A	133549137	0.0202	nonsynonymous SNV	NM_001015877	PHF6	p.R274Q
P108	chr4	106157539	c.2440C>T	106157539	0.4829	nonsynonymous SNV	NM_001127208	TET2	p.R814C

P108	chr21	44524456	c.101C>T	44524456	0.438	nonsynonymous SNV	NM_006758	U2AF1	p.S34F
P109	chr2	25466785	c.1917_1918del	25466786	0.9456	frameshift deletion	NM_022552	DNMT3A	p.L639fs
P109	chr2	209113112	c.395G>T	209113112	0.432	nonsynonymous SNV	NM_001282386	IDH1	p.R132L
P109	chr7	82581549	c.8720G>C	82581549	0.5029	nonsynonymous SNV	NM_033026	PCLO	p.R2907T
P110	chr2	25467164	c.1711delG	25467164	0.0081	frameshift deletion	NM_022552	DNMT3A	p.A571fs
P110	chr11	32417919	c.1132_1133del	32417920	0.4983	frameshift deletion	NM_024426	WT1	p.L378fs
P110	chr11	32410620	c.1534_1538del	32410624	0.027	frameshift deletion	NM_024426	WT1	p.K512fs
P110	chr11	32410614	c.1544delT	32410614	0.027	frameshift deletion	NM_024426	WT1	p.L515fs
P110	chr11	32410617	c.1540_1541insT	32410617	0.0275	frameshift insertion	NM_024426	WT1	p.Q514fs
P110	chr13	41240044	c.298_306del	41240052	0.4883	nonframeshift deletion	NM_002015	FOXO1	p.100_102del
P110	chr8	128750682	c.219_220insCCG	128750682	0.0121	nonframeshift insertion	NM_002467	MYC	p.T73delinsTP
P110	chr2	209113112	c.395G>A	209113112	0.4129	nonsynonymous SNV	NM_001282386	IDH1	p.R132H
P110	chr11	32417914	c.1138C>G	32417914	0.5049	nonsynonymous SNV	NM_024426	WT1	p.R380G
P110	chr11	32410616	c.1542G>T	32410616	0.0271	nonsynonymous SNV	NM_024426	WT1	p.Q514H
P110	chr7	66459197	c.258+2T>C	66459197	0.4637	Splice_Site	NM_016038	SBDS	-
P111	chr7	148506170	c.2172dupT	148506170	0.071	stopgain	NM_001203247	EZH2	p.D725_Y726delins X
P111	chr6	157100097	c.1034C>T	157100097	0.4774	nonsynonymous SNV	NM_017519	ARID1B	p.A345V
P111	chr2	48030591	c.3205G>C	48030591	0.5033	nonsynonymous SNV	NM_000179	MSH6	p.G1069R
P112	chr10	27318266	c.3824G>A	27318266	0.3828	nonsynonymous SNV	NM_001256053	ANKRD26	p.R1275Q
P112	chr2	25966159	c.3047C>T	25966159	0.4872	nonsynonymous SNV	NM_018263	ASXL2	p.T1016M
P112	chr7	2979559	c.688G>A	2979559	0.034	nonsynonymous SNV	NM_032415	CARD11	p.D230N
P112	chr2	25457242	c.2645G>A	25457242	0.3934	nonsynonymous SNV	NM_022552	DNMT3A	p.R882H
P112	chr2	209113113	c.394C>T	209113113	0.375	nonsynonymous SNV	NM_001282386	IDH1	p.R132C
P112	chr15	88680721	c.536A>G	88680721	0.0073	nonsynonymous SNV	NM_001012338	NTRK3	p.E179G
P112	chr1	215821895	c.14557A>G	215821895	0.4874	nonsynonymous SNV	NM_206933	USH2A	p.M4853V
P112	chr4	187535372	c.9202G>T	187535372	0.3712	stopgain	NM_005245	FAT1	p.E3068X
P113	chr4	55599321	c.2447A>T	55599321	0.428	nonsynonymous SNV	NM_000222	KIT	p.D816V
P113	chr1	39854112	c.9412A>G	39854112	0.0055	nonsynonymous SNV	NM_012090	MACF1	p.R3138G
P113	chr4	106156630	c.1531C>T	106156630	0.5092	nonsynonymous SNV	NM_001127208	TET2	p.H511Y
P113	chr17	48762146	c.4190G>A	48762146	0.5132	stopgain	NM_003786	ABCC3	p.W1397X
P114	chrX	133511776	c.130dupA	133511776	0.0604	frameshift insertion	NM_001015877	PHF6	p.H43fs
P114	chrX	39913229	c.4784A>G	39913229	1	nonsynonymous SNV	NM_001123383	BCOR	p.D1595G
P114	chr18	42530144	c.839A>G	42530144	0.4784	nonsynonymous SNV	NM_015559	SETBP1	p.N280S
P114	chr2	39283913	c.440A>T	39283913	0.031	nonsynonymous SNV	NM_005633	SOS1	p.N147I
P114	chr21	44524453	c.104G>A	44524453	0.0396	nonsynonymous SNV	NM_006758	U2AF1	p.R35Q
P114	chr1	43818443	c.1908A>G	43818443	0.4856	stoploss	NM_005373	MPL	p.X636W
P115	chr4	55589767	c.1249_1256delinsTTCGG	55589774	0.3196	frameshift deletion	NM_000222	KIT	p.T417_D419delinsF R
P115	chr2	25973137	c.1287dupA	25973137	0.3644	frameshift insertion	NM_018263	ASXL2	p.E430fs
P115	chr1	115258747	c.35G>A	115258747	0.0121	nonsynonymous SNV	NM_002524	NRAS	p.G12D
P115	chrX	70466326	c.2413A>C	70466326	0.0615	nonsynonymous SNV	NM_001171162	ZMYM3	p.T805P
P115	chr17	29679366	c.7486C>T	29679366	0.3714	stopgain	NM_000267	NF1	p.R2496X
P116	chr19	33793207	c.105_114del	33793216	0.4665	frameshift deletion	NM_004364	CEBPA	p.R35fs
P116	chr13	28608260	c.1796_1802delinsCGGA	28608256	0.0932	nonframeshift deletion	NM_004119	FLT3	p.Y599_L601delinsS D

P116	chr19	33792381	c.939_940insAAG	33792381	0.4691	nonframeshift insertion	NM_004364	CEBPA	p.V314delinsKV
P116	chr7	101892095	c.4291_4292insGCCCCGCGGCCCCGA	101892095	0.3944	nonframeshift insertion	NM_181552	CUX1	p.G1431delinsGPAAPS
P116	chr13	28608250	c.1805_1806insGGTTGATTTCAGAGAATATGAATATGATCTCAA	28608250	0.2777	nonframeshift insertion	NM_004119	FLT3-ITD	p.K602delinsKVDFREYEYDLK
P116	chr7	148512600	c.1529A>G	148512600	0.0077	nonsynonymous SNV	NM_001203247	EZH2	p.K510R
P116	chr1	115258748	c.34G>A	115258748	0.163	nonsynonymous SNV	NM_002524	NRAS	p.G12S
P116	chr1	115258745	c.37G>C	115258745	0.0232	nonsynonymous SNV	NM_002524	NRAS	p.G13R
P116	chr7	82763933	c.2933G>A	82763933	0.4969	nonsynonymous SNV	NM_033026	PCLO	p.G978E
P116	chr22	41556727	c.3671+1G>A	41556727	0.4633	Splice_Site	NM_001429	EP300	-
P117	chr19	33793180	c.135_141del	33793186	0.4206	frameshift deletion	NM_004364	CEBPA	p.P45fs
P117	chr7	148504768	c.2211delC	148504768	0.2358	frameshift deletion	NM_001203247	EZH2	p.V737fs
P117	chr19	33792400	c.920_921insGCGCAA	33792400	0.4387	nonframeshift insertion	NM_004364	CEBPA	p.N307delinsKRN
P117	chr6	157100063	c.1000G>C	157100063	0.4932	nonsynonymous SNV	NM_017519	ARID1B	p.A334P
P117	chr7	148544291	c.100C>T	148544291	0.5617	stopgain	NM_001203247	EZH2	p.R34X
P118	chr19	33792995	c.326delC	33792995	0.4125	frameshift deletion	NM_004364	CEBPA	p.P109fs
P118	chr7	50467674	c.909_910del	50467675	0.4001	frameshift deletion	NM_006060	IKZF1	p.N303fs
P118	chr2	25966113	c.3093G>C	25966113	0.5152	nonsynonymous SNV	NM_018263	ASXL2	p.Q1031H
P118	chr19	33792458	c.863G>C	33792458	0.4066	nonsynonymous SNV	NM_004364	CEBPA	p.R288P
P118	chr16	3778287	c.6761T>C	3778287	0.0051	nonsynonymous SNV	NM_004380	CREBBP	p.L2254P
P118	chr4	55599321	c.2447A>T	55599321	0.036	nonsynonymous SNV	NM_000222	KIT	p.D816V
P118	chr1	115256528	c.183A>T	115256528	0.008	nonsynonymous SNV	NM_002524	NRAS	p.Q61H
P118	chr1	115258747	c.35G>A	115258747	0.1812	nonsynonymous SNV	NM_002524	NRAS	p.G12D
P118	chr10	112343964	c.1115G>C	112343964	0.4	nonsynonymous SNV	NM_005445	SMC3	p.R372T
P118	chr5	1293998	c.1003T>C	1293998	0.4922	nonsynonymous SNV	NM_198253	TERT	p.S335P
P119	chr2	25972852	c.1572_1573insAGGTTCCG	25972852	0.4653	frameshift insertion	NM_018263	ASXL2	p.P525fs
P119	chrX	44942750	c.3486_3487insCTCC	44942750	0.0202	frameshift insertion	NM_001291415	KDM6A	p.V1162fs
P119	chrX	133511707	c.59_60insTGATATTC	133511707	0.036	frameshift insertion	NM_001015877	PHF6	p.C20fs
P119	chr4	55589768	c.1248_1257delinsTTTCGGA	55589775	0.12035	nonframeshift deletion	NM_000222	KIT	p.L416_D419delinsLFG
P119	chr12	49445379	c.1899_2087del	49445567	0.0061	nonframeshift deletion	NM_003482	KMT2D	p.633_696del
P119	chr4	55599321	c.2447A>T	55599321	0.2107	nonsynonymous SNV	NM_000222	KIT	p.D816V
P119	chr4	55599340	c.2466T>A	55599340	0.0799	nonsynonymous SNV	NM_000222	KIT	p.N822K
P119	chr1	39851517	c.8074A>C	39851517	0.5172	nonsynonymous SNV	NM_012090	MACF1	p.I2692L
P120	chr7	50444323	c.253_260del	50444330	0.0382	frameshift deletion	NM_006060	IKZF1	p.L85fs
P120	chr19	33793203	c.117dupC	33793203	0.4754	frameshift insertion	NM_004364	CEBPA	p.A40fs
P120	chr4	55589767	c.1251_1256delinsGGG	55589774	0.018233333	nonframeshift deletion	NM_000222	KIT	p.T417_D419delinsTG
P120	chr19	33792347	c.973_974insTGGAGCTTACCAGTGACAATGACCGCCTGC	33792347	0.4798	nonframeshift insertion	NM_004364	CEBPA	p.R325delinsLELTSDNDRLR
P120	chr13	28608248	c.1807_1808insTAAGAGAATATGAATATGATCTCAAAT	28608248	0.1998	nonframeshift insertion	NM_004119	FLT3-ITD	p.W603delinsLREYEYDLKW
P120	chr16	3779619	c.5429G>A	3779619	0.0053	nonsynonymous SNV	NM_004380	CREBBP	p.R1810H
P120	chr16	3778287	c.6761T>C	3778287	0.006	nonsynonymous SNV	NM_004380	CREBBP	p.L2254P
P120	chr1	36933434	c.1853C>T	36933434	0.0388	nonsynonymous SNV	NM_156039	CSF3R	p.T618I
P120	chr4	55599321	c.2447A>T	55599321	0.1639	nonsynonymous SNV	NM_000222	KIT	p.D816V
P120	chr12	25398284	c.35G>T	25398284	0.0263	nonsynonymous SNV	NM_004985	KRAS	p.G12V

P120	chr1	36932107	c.2442dupT	36932107	0.0134	stopgain	NM_156039	CSF3R	p.E815_N816delins X

* It was found that 99.2% (120/121) of AML patients had at least one mutation detected by targeted NGS.

Supplementary Table S5. Univariate analysis of clinical characteristics in 121 patients with Intermediate-risk AML.

Factors	Overall survival			Relapse-free survival		
	HR	95% CI	*P*	HR	95% CI	*P*
Age, (years), ＜55 *vs.* ≥55	2.13	1.190-3.811	**0.011**	2.035	1.125-3.680	**0.019**
Sex, male *vs.* female	0.79	0.462-1.352	0.39	1.065	0.634-1.788	0.811
WBC count (×10^9/L), ＜10 *vs.* ≥10	2.153	1.217-3.809	**0.008**	2.038	1.169-3.555	**0.012**
PLT count (×10^9/L) ≤40 *vs.* ＞40	1.765	1.001-3.113	**0.049**	1.105	0.632-1.932	0.727
Hb (g/L)	1.692	0.921-3.107	0.09	1.352	0.734-2.491	0.333
LDH (U/L)	1.001	1.000-1.001	**0.018**	1	1.000-1.001	0.213
MRD after cycle1 (%), ＜0.1 *vs.* ≥0.1	0.959	0.556-1.654	0.88	0.908	0.532-1.548	0.723
MRD after cycle2 (%), ＜0.1 *vs.* ≥0.1	0.938	0.522-1.685	0.829	0.981	0.552-1.741	0.947
MRD before post remission chemotherapy (%), ＜0.1 *vs.* ≥0.1	0.995	0.512-1.935	0.988	1.205	0.652-2.227	0.552
ECOG at diagnosis, ≤1 *vs.* 2 *vs.* 3	1.373	0.892-2.114	0.149	1.387	0.905-2.124	0.133
the cycles of induction to obtain CR, 1-2 cycles *vs.* ≥ 3 cycles	1.705	0.761-3.820	0.195	1.386	0.626-3.067	0.42
Treatment, alloHSCT *vs.* PR-CT	1.802	1.008-3.222	**0.047**	2.067	1.183-3.6110	**0.011**
Mutations, no *vs.* yes						
FLT3-ITD	1.696	0.924-3.112	0.088	1.924	1.080-3.428	**0.026**
DNMT3A	2.564	1.455-4.519	**0.001**	2.138	1.195-3.824	**0.01**
IDH2	0.378	0.118-1.213	0.102	0.597	0.238-1.497	0.272
CEBPA	0.866	0.466-1.611	0.65	0.935	0.519-1.684	0.823
ARID1B	0.981	0.394-2.439	0.966	0.951	0.387-2.334	0.914
KIT	0.966	0.527-1.769	0.91	0.942	0.523-1.695	0.841
NRAS	1.172	0.648-2.119	0.599	1.199	0.681-2.113	0.529
Mutations in signaling Pathway, no *vs.* yes	2.477	1.169-5.251	**0.018**	1.848	0.933-3.661	0.078
Risk score, <1 *vs.* ≥1	4.427	2.299-8.524	<0.0001	3.103	1.724-5.585	<0.0001

HR, hazards ratio; CI, confidence interval; WBC, white blood cell count; LDH, lactate dehydrogenase, MRD, minimal residual disease; ECOG, Eastern Cooperative Oncology Group; CR, complete remission; PLT, platelet; HSCT, hematopoietic stem cell transplantation

Supplementary Table S6. Multivariate analysis of clinical characteristics in 121 patients with Intermediate-risk AML.

Variables	Overall survival			Relapse-free survival		
	HR	95% CI	*P*	HR	95% CI	*P*
Age (years), ＜55 *vs.* ≥55	-	-	-	-	-	-
WBC count (×10^9/L) , ＜10 *vs.* ≥10	2.187	1.224- 3.907	0.0082	2.139	1.203- 3.804	0.0096
PLT count (×10^9/L) , ≤40 *vs.* > 40	-	-	-	-	-	-
LDH (U/L)	-	-	-	-	-	-
Mutations, no vs. yes						
FLT3-ITD	-	-	-	-	-	-
DNMT3A	3.043	1.675- 5.527	0.0003	2.316	1.214- 4.417	0.0108
Mutations in signaling Pathway, no *vs.* y	1.962	1.003- 3.840	0.0496	-	-	-

HR, hazards ratio; CI, confidence interval; WBC, white blood cell count; LDH, lactate dehydrogenase, PLT, platelet

Supplementary Table S7. The characteristics of DNMT3A^{neg} and DNMT3A^{pos} in intermediate risk AML patients ≤65 years old

Parameters	DNMT3A^{neg}	DNMT3A^{pos}	*P*
Total cohort	93	28	
Sex , *n* (%)			0.601
Males	55 (59.1)	15 (53.6)	
Females	38 (40.9)	13 (46.4)	
Median age (range), years	41 (14-65)	47 (20-65)	0.009
Laboratory parameters			
WBC count, median (range) ×10^9/L	11.2 (0.5-207.7)	17.1 (0.7-210.1)	0.276
Platelet count, median (range) ×10^9/L	30.0 (4.0-561.0)	91.5 (6.0-197.0)	0.011
Hemoglobin count, median (range) ×10^9/L	89.0 (42.0-204.0)	78.0 (44.0-163.0)	0.878
BM blast, median (range) %	68.0 (21.0-96.5)	61.2 (21.0-98.0)	0.115
LDH, median (range) U/L	314.0 (61.0-2567.0)	227.0 (97.0-1644.0)	0.212
Flow cytometry-MRD before PRT, median (range) %	0.1 (0.0-12.0)	0.1 (0.0-3.9)	0.863
ECOG Performance Status at Diagnosis, *n* (%)			0.315
≤1	62 (66.7)	15 (53.6)	
2	26 (27.9)	12 (42.8)	
3	5 (5.4)	1 (3.6)	
Cytogenetics, *n* (%)			0.803
Normal karyotype	49 (52.7)	14 (50.0)	
Other	44 (37.6)	14 (35.7)	
CR reached after			0.211
Cycle1 (early CR)	59(45.2)	15 (64.3)	
Cycle2 (late CR)	19 (14.0)	4 (21.4)	
Other	15 (40.8)	9 (14.3)	
Flow cytometry-MRD before PRT			0.079
MRD^{pos*}(≥ 0.1%)	20 (21.5)	5 (17.9)	
MRD^{neg*}(< 0.1%)	61 (65.6)	14 (50.0)	
Miss	12 (12.9)	9 (32.1)	
Hematopoietic stem cell transplantation, *n* (%)			0.24
No	55 (59.1)	20 (71.4)	

Yes	38 (40.9)	8 (28.6)	
Relapse, *n* (%)			0.562
No	62 (66.7)	17 (60.7)	
Yes	31 (33.3)	11 (39.3)	
Death, *n* (%)			0.029
No	55 (59.1)	10 (35.7)	
Yes	38 (40.9)	18 (64.3)	
Treatment, *n* (%)			0.007
DA	83 (89.2)	18 (64.3)	
D-CAG	6 (6.5)	6 (21.4)	
Other	4 (4.3)	4 (14.3)	
3-year OS, %	54.8±5.7	24.0±9.3	0.001
3-year RFS, %	46.5±5.9	21.2±9.3	0.009
Follow-up; median (range) months	38.1 (1.0-102.2)	9.0 (4.1-87.6)	0.084

Abbreviations: WBC, white blood cell count; BM, bone marrow; LDH, lactate dehydrogenase, MRD, minimal residual disease before cycle1, cycle2 or before post remission chemotherapy; ECOG, Eastern Cooperative Oncology Group, AML, acute myeloid leukemia; FAB, French-American British classification; NOS, non-specific type; CR, complete remission; DA, daunorubicin +cytarabine; D-CAG, decitabine combined with low-dose arabinosylcytosine (Ara-c), aclarubicin and granulocyte colony-stimulating factor (G-CSF); OS, overall survival (with the event of death, regardless of the cause); RFS, relapse-free survival (with the event of death in first CR or relapse)

* MRD^{neg}, MRD ＜0.1%; MRD^{pos}, MRD ≥0.1%.

Supplementary Figures

Supplementary Figure S1

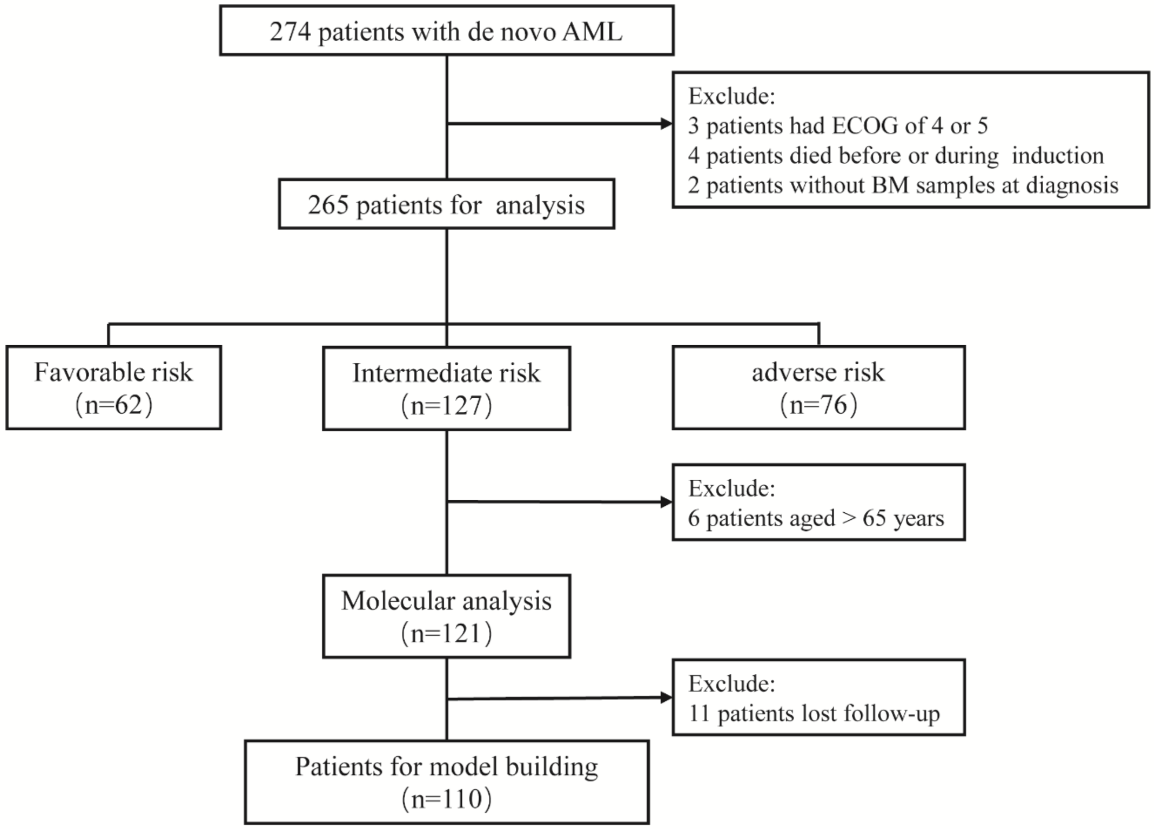

Supplementary Figure S1. Flow chart of analysis.

Supplementary Figure S2

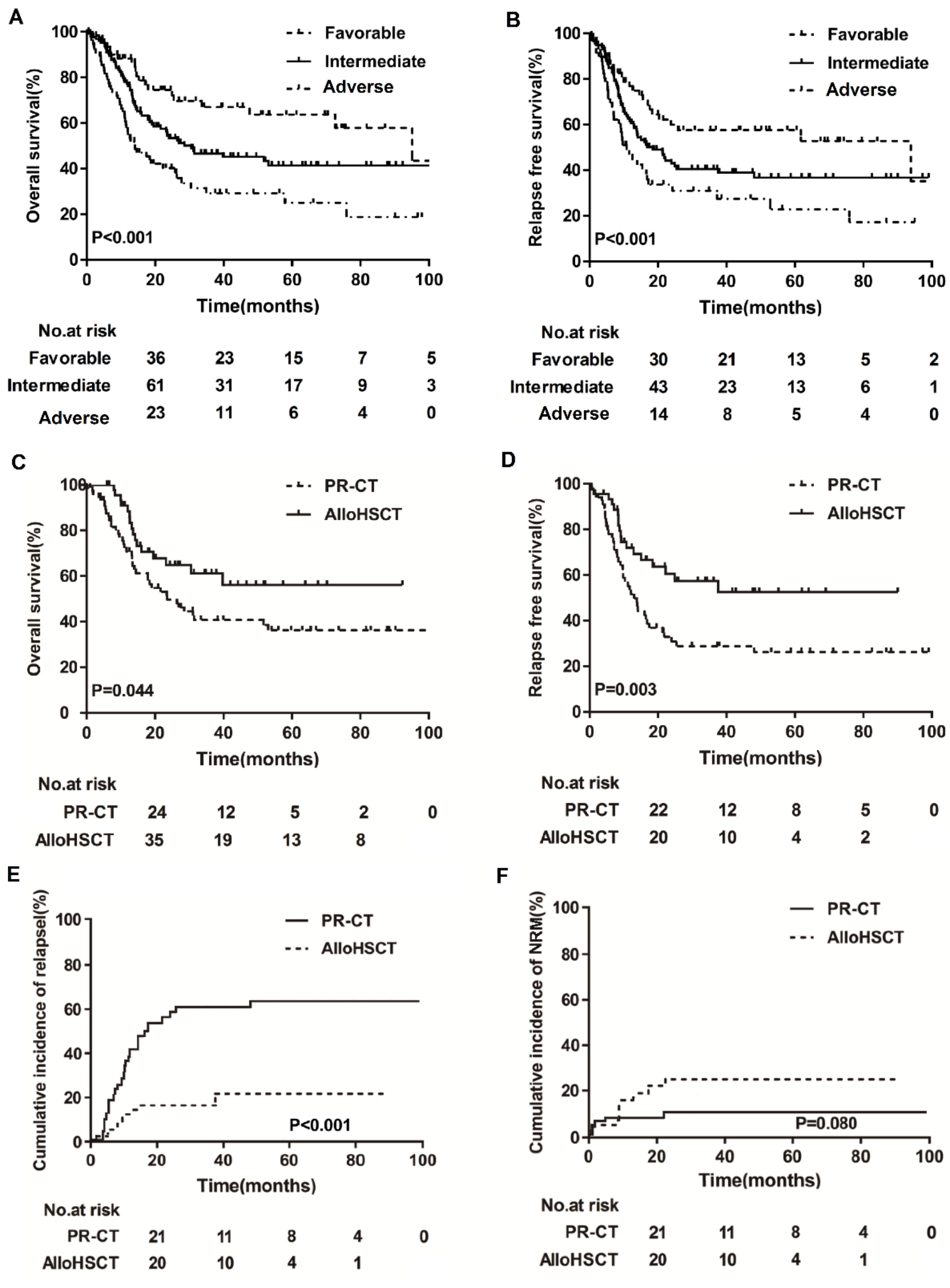

Supplementary Figure S2 OS (A) and RFS (B) for patients with ELN defined favorable, intermediate, and adverse AML (n=256). OS(C), RFS(D), CIR (E) and NRM (F) were for patients aged 14- 65 years (n=121) who received alloHSCT (n=46) or PR-CT (n=75), respectively.

Supplementary Figure S3

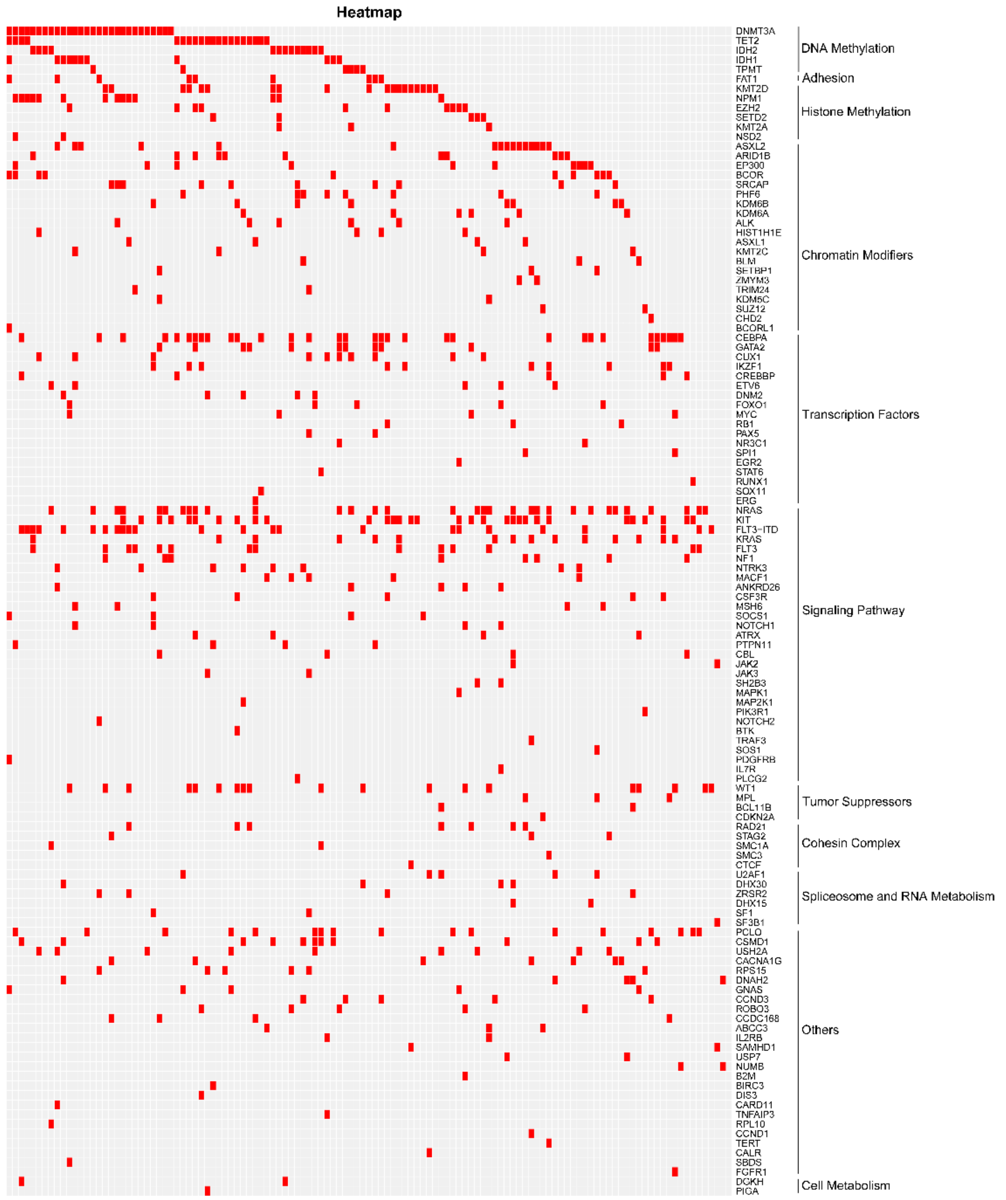

Supplementary Figure S3 (A) Mutation frequencies based on functional classification.

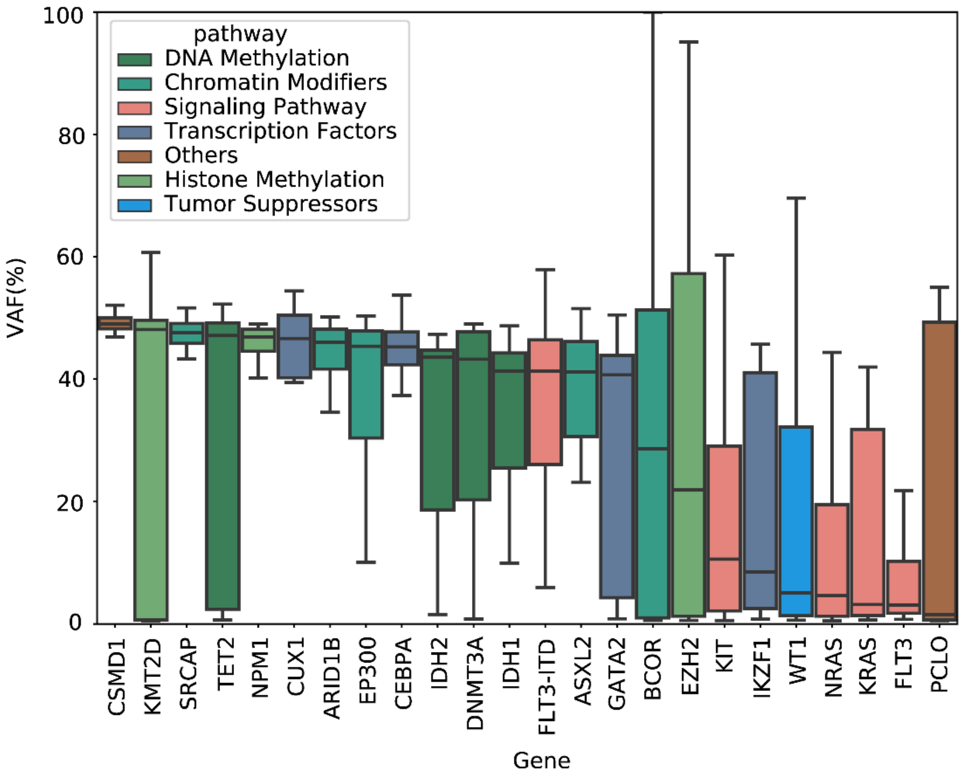

Supplementary Figure S3 (B) Analysis of variant allele frequencies (VAFs) of the mutated genes in ≥10 patients. The boxplot shows the median, 25th, and 75th percentiles. VAFs were adjusted to a copy number. The boxes are colored in accordance with the functional category assigned to each gene.

Supplementary Figure S4

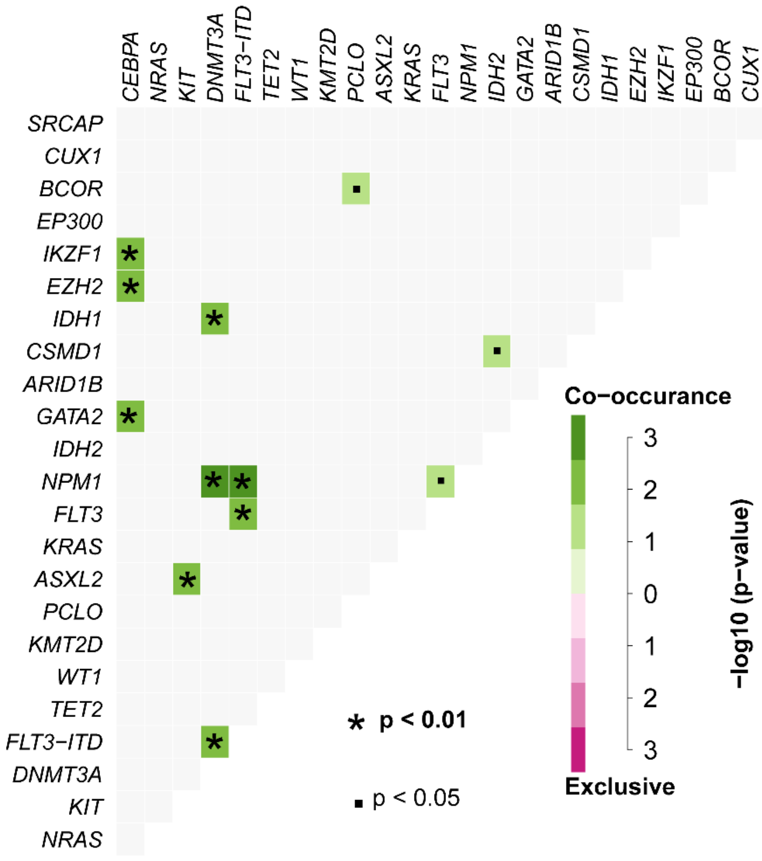

Supplementary Figure S4 Co-occurrence and mutually exclusive mutations among 121 intermediate-risk AML patients. Green color indicates a positive associate, and purple color indicates a negative association.

Supplementary Figure S5

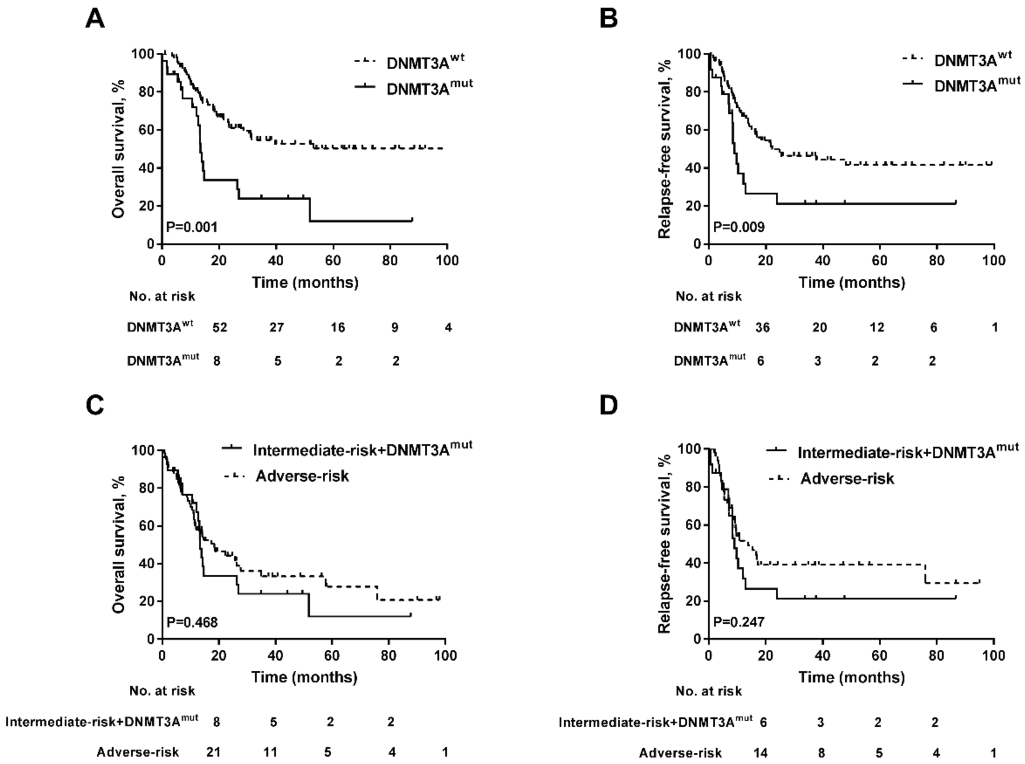

Supplementary Figure S5 The Kaplan-Meier survival curves for OS and RFS for patients with and without *DNMT3A* mutation, respectively. (A) OS, (B) RFS. Survival of intermediate-risk patients with *DNMT3A* mutation (n=28), and adverse-risk patients. (C) OS, (D) RFS.

Supplementary Figure S6

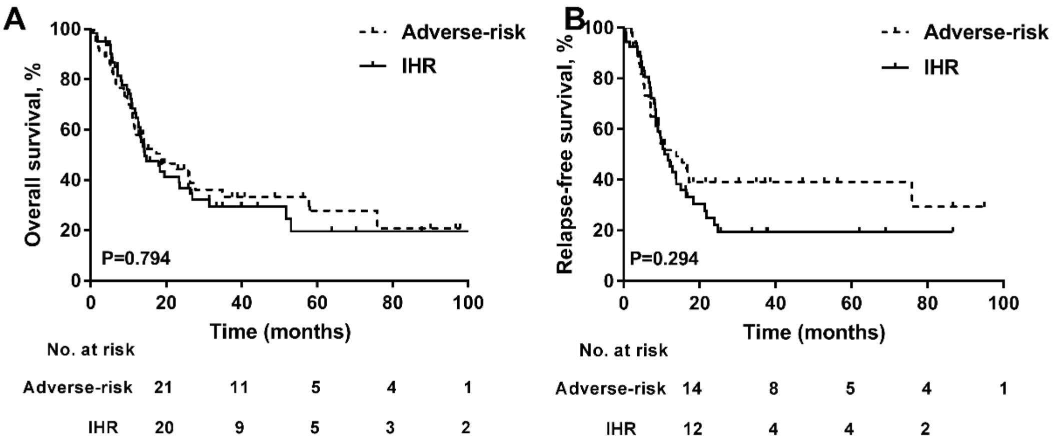

Supplementary Figure S6 Kaplan-Meier curves for OS and RFS in 2017 ELN adverse-risk AML patients and IHR AML, respectively. (A) OS; (B) RFS.
